# Muscle Synergies in Response to Biofeedback-Driven Gait Adaptations in Children With Cerebral Palsy

**DOI:** 10.3389/fphys.2019.01208

**Published:** 2019-09-27

**Authors:** Adam T. C. Booth, Marjolein M. van der Krogt, Jaap Harlaar, Nadia Dominici, Annemieke I. Buizer

**Affiliations:** ^1^Department of Rehabilitation Medicine, Amsterdam Movement Sciences, Amsterdam UMC, Vrije Universiteit Amsterdam, Amsterdam, Netherlands; ^2^Department of Clinical Applications and Research, Motek Medical BV, Amsterdam, Netherlands; ^3^Department of Biomechanical Engineering, Delft University of Technology, Delft, Netherlands; ^4^Department of Human Movement Sciences, Faculty of Behavioural and Movement Sciences, Institute for Brain and Behavior Amsterdam & Amsterdam Movement Sciences, Vrije Universiteit Amsterdam, Amsterdam, Netherlands

**Keywords:** motor control, EMG, feedback, virtual reality, rehabilitation

## Abstract

**Background:**

Children with cerebral palsy (CP) often show impaired selective motor control (SMC) that induces limitations in motor function. Children with CP can improve aspects of pathological gait in an immediate response to visual biofeedback. It is not known, however, how these gait adaptations are achieved at the neural level, nor do we know the extent of SMC plasticity in CP.

**Aim:**

Investigate the underlying SMC and changes that may occur when gait is adapted with biofeedback.

**Methods:**

Twenty-three ambulatory children with CP and related (hereditary) forms of spastic paresis (Aged: 10.4 ± 3.1, 6–16 years, M: 16/F: 9) were challenged with real-time biofeedback to improve step length, knee extension, and ankle power while walking on an instrumented treadmill in a virtual reality environment. The electromyograms of eight superficial muscles of the leg were analyzed and synergies were further decomposed using non-negative matrix factorization (NNMF) using 1 to 5 synergies, to quantify SMC. Total variance accounted for (tVAF) was used as a measure of synergy complexity. An imposed four synergy solution was investigated further to compare similarity in weightings and timing patterns of matched paired synergies between baseline and biofeedback trials.

**Results:**

Despite changes in walking pattern, changes in synergies were limited. The number of synergies required to explain at least 90% of muscle activation increased significantly, however, the change in measures of tVAF_1_ from baseline (0.75 ± 0.08) were less than ±2% between trials. In addition, within-subject similarity of synergies to baseline walking was high (>0.8) across all biofeedback trials.

**Conclusion:**

These results suggest that while gait may be adapted in an immediate response, SMC as quantified by synergy analysis is perhaps more rigidly impaired in CP. Subtle changes in synergies were identified; however, it is questionable if these are clinically meaningful at the level of an individual. Adaptations may be limited in the short term, and further investigation is essential to establish if long term training using biofeedback leads to adapted SMC.

## Introduction

Walking requires refined coordination of muscle activation. The current theory in motor control suggests the central nervous system acts to simplify this complexity by the recruitment of a small number of muscle synergies (Tresch et al., 2002). A muscle synergy, or module, is the balanced temporal activation of a group of muscles to create a specific movement. These patterns have also been described as primitives due to their nature as building blocks for movement and because they are linked to ancestral generation of locomotion, observed across a number of animal species (Dominici et al., 2011).

Understanding these fundamental motor control strategies may help to develop effective treatments for individuals with impaired motor function. In individuals with neurologic impairment, such as cerebral palsy (CP), selective motor control (SMC) is often affected. SMC has been defined as “the ability to isolate the activation of muscles in a selected pattern in response to demands of a voluntary movement or posture” (Sanger et al., 2006). Impaired SMC has been linked to damage in the corticospinal tract (Fowler et al., 2010), limiting the ability of an individual to modulate muscle activation required for an efficient walking pattern. Impaired SMC can be quantified by clinical measures such as the selective control assessment of the lower extremities (Fowler et al., 2009) and has a direct relationship with the level of gait impairment (Chruscikowski et al., 2017). Muscle synergy analysis has been used to quantify the impairment of SMC during gait analysis in children with CP (Steele et al., 2015; Tang et al., 2015; Schwartz et al., 2016). Using non-negative matrix factorization (NNMF) of measured surface electromyography (EMG), it has been shown that a few muscle synergy patterns can describe over 90% of measured muscle activity, or total variance accounted for (tVAF), in the majority of typically developing (TD) individuals during walking (Steele et al., 2015; Cappellini et al., 2016, 2018). Most individuals with CP show a reduced number of synergies compared to TD children (Steele et al., 2015), indicating a different motor control strategy. These methods to quantify SMC have been linked to success of treatment outcomes across a range of treatment strategies (Schwartz et al., 2016; Shuman et al., 2018).

The extent of adaptability and plasticity of muscle synergies during walking is not fully understood. Synergies are considered the base unit of control for movement. If synergies are unchanging neural circuits, this may imply that little can be done to train improved motor functions in individuals with impaired SMC during walking. The developing child brain shows high plasticity. The number and complexity of muscle synergies evolve with age, and distinct changes can be discerned from neonate, to toddler, to adult (Dominici et al., 2011). This plasticity may be targeted in children with neurologic impairment. If synergies can be adapted, it may present a promising strategy for rehabilitation.

It is often considered that children with CP have limited potential to adapt gait pattern with conservative treatment; however, with real-time biofeedback, children with CP are able to improve aspects of gait in an immediate response (Baram and Lenger, 2012; van Gelder et al., 2017; Booth et al., 2019). While they can achieve this change in gait, it is not known how this is achieved at the neural level. Gait training was found to subtly influence synergy composition and timing in some individuals post-stroke, which was associated with improved walking performance (Routson et al., 2013). It may be that to reach a more typical gait pattern, recruitment of a more complex synergistic strategy is required. If it is possible to adapt synergy patterns with biofeedback, this might form a promising technique to train SMC with the goal of improving gait. Furthermore, those with greater synergistic complexity may show increased capacity to adapt gait with training. Targeting SMC plasticity in children with neurological impairment could have large implications on the long-term effectiveness of treatment interventions.

Therefore, the aim of this study was to establish if SMC, as quantified using synergy analysis during gait, is changed when children with CP are challenged to improve aspects of gait with real-time biofeedback. A secondary aim was to assess whether children showing greater gait improvements in response to biofeedback have less impaired SMC than those with more limited adaptability of gait.

## Materials and Methods

### Participants

Twenty-five children with CP and related (hereditary) forms of spastic paresis were recruited in this study. Children were included under the following criteria: diagnosis of spastic paresis (unilateral and bilateral), walking without aids – for CP gross motor function classification system (GMFCS) level I-II (Palisano et al., 1997) – and aged between 5 and 16 years old. Children were excluded if they had severe cognitive or visual impairment; had received botulinum toxin-A treatment within the previous 6 months; or had orthopedic surgery, intrathecal baclofen treatment, or selective dorsal rhizotomy within 12 months prior to measurement date. Children were recruited from the VU University Medical Center, Amsterdam and Revant Rehabilitation Center, Breda. In addition, 27 TD children (aged 10.0 ± 2.11, range: 5–16 years) were included to provide reference normative data for treadmill walking. All parents and children aged 12 years and older provided written informed consent prior to participation. The protocol was approved by the medical ethics committee of the VU University Medical Center (NL56736.029.1). The kinematic and kinetic analysis of this dataset is presented in a separate manuscript (Booth et al., 2019).

### Study Design

**FIGURE 1:**
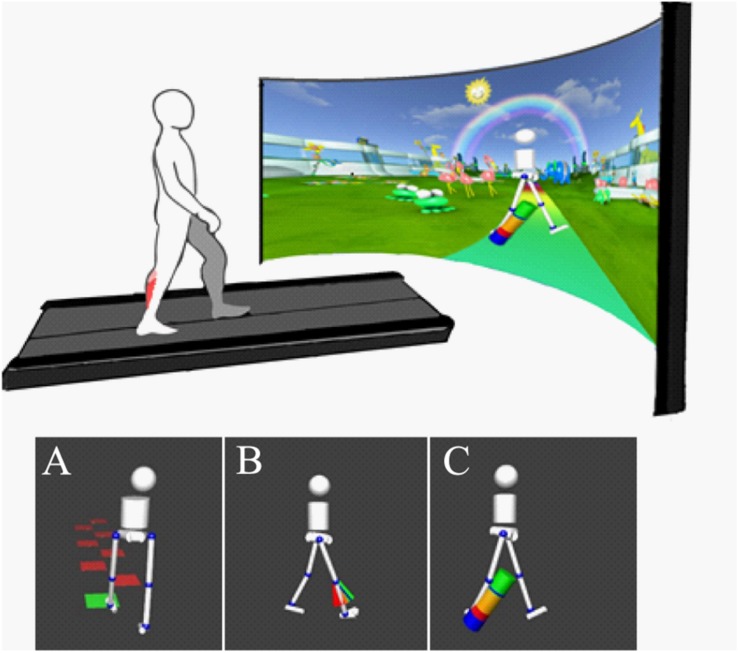
**Top:** Experimental setup with treadmill with dual force plates and virtual reality screen (Motek Medical, Amsterdam). 3D motion capture (Vicon, Oxford) is integrated with virtual reality using D-Flow software (Motek Medical, Amsterdam) to provide real-time gait analysis and visualization. **Bottom:** Visual biofeedback presented, background environment removed from image in figure for clarity. Biofeedback involved visualization of movement in real time using simplified avatar. Color-coded biofeedback was attached to an avatar providing feedback on (A) step length (goal to step on the blocks), (B) knee extension (goal to reach the green area of “fan,” showing knee extension in swing), and (C) ankle power (goal to increase “power-bar” at the ankle during push-off).

All participants walked on an instrumented treadmill with an immersive virtual reality environment (GRAIL, Motek Medical, Amsterdam, Netherlands. Figure 1). A 10-camera 3D motion capture (Vicon, Oxford, United Kingdom) system was used with 26 retroreflective markers placed on anatomical landmarks. This allowed for real-time gait analysis using the human body model (van den Bogert et al., 2013; Falisse et al., 2018). A non-weight bearing safety harness was worn in all conditions to prevent injury in case of accidental slip.

Following a period of at least 6 min habituation to walking on the treadmill, self-selected comfortable walking speed was fixed. This speed was maintained throughout the session. Participants initially carried out 1 min of comfortable walking. Baseline gait function was used to set individual targets for subsequent biofeedback trials. Targets were adapted during trials based on individual response to maintain motivation. Participants performed a series of trials, in a randomized order, lasting 2 min, in which they were challenged to improve gait in response to avatar-based real-time biofeedback on step length, knee extension during late swing and ankle push off power (Figure 1). Feedback on ankle power and knee extension was provided on the most affected side only and visualized only during the target phase of the gait cycle. Most impaired limb was either indicated by the clinician or analysis of the child’s baseline walking to assess which had the greatest limitation from typical walking. These specific targets for biofeedback were chosen as they were considered commonly observed clinically relevant gait parameters of different nature (spatiotemporal, kinematic and kinematic parameters, respectively).

### Electromyography

Surface electromyography data (MiniWave, Cometa, Italy) were collected at 1000 Hz on 8 muscles of the lower leg: gluteus medius, rectus femoris, vastus lateralis, semitendinosus, tibialis anterior, gastrocnemius medialis, soleus, and peroneus longus muscles following SENIAM guidelines (Hermens et al., 2000). For each individual, only the most involved limb, on which biofeedback was provided, was used for analysis. Initial contact and toe-off instances were based on vertical ground reaction forces and marker data (Zeni et al., 2008). EMG data were digitally processed for analysis in the following sequence: (1) band-pass filtered at 20–450 Hz, (2) full wave rectified, and (3) low-pass filtered at 10 Hz to obtain a linear envelope. For all filtering steps, bidirectional 6th order Butterworth filters were used to prevent time drift. EMG envelopes for each muscle were time-normalized to gait cycles. Signals of all trials were normalized to mean dynamic activation of each muscle during the baseline walking trial. To reduce within-subject variability, yet maximize number of steps included, a random sample of the minimum number of strides available from any trial across each participant was concatenated, providing a synergy input matrix of 8×(101×*minimumno*.*strides*)per walking trial. On average 34.4 ± 9.7 strides were concatenated for analysis (range: 13–49 strides).

### Synergy Analysis

Muscle synergies during walking were quantified as a measure for SMC using NNMF (MATLAB v2017a, MathWorks, MA, United States) with 100 max iterations and 1000 replicates. NNMF is a widely used mathematical algorithm for condensing measured muscle activation into sets of synergistic action (Tang et al., 2015; Shuman et al., 2017).

Each muscle activation can be best described by the synergy timing co-efficient (C) and the relative weighting (W) of each muscle toward this synergy timing, with an additional error of the reconstructed signal, following:

Muscle⁢Activation=W×C+e⁢r⁢r⁢o⁢r

where W is a *m × n* matrix with *m* the number of muscles (eight) and *n* the specified number of synergies (from one to five in this analysis). C is an *n × t* matrix where *t* is the number of time points. To maximize data available for analysis, in the event of missing or erroneous EMG signals, this channel was given zero weight and not incorporated into the NNMF, i.e., NNMF for the trial analysis would then be calculated for 7 muscles (Shuman et al., 2018). How well the extracted synergies describe measured EMG activity was calculated by the tVAF at each synergy solution (Ting and Macpherson, 2005), such that tVAF = 1 -*SSE*/SST, where the sum squared of errors (SSE) is the unexplained variation and total sum of squares (SST) is the pooled variation of data. No thresholds for synergy computation were applied. The minimum number of synergies required to describe at least 90% of tVAF of the measured EMG was defined as an indicative threshold for redundancy, following (Clark et al., 2010; Oliveira et al., 2014; Rozumalski et al., 2017).

#### Similarity of Synergies

To allow for comparison of synergy structures and weightings between individuals and trials, we further investigated the four-synergy level solution. From preliminary analysis, four synergies were sufficient to describe at least 90% tVAF in the majority of TD participants. Therefore, by exploring the four-synergy solution in greater detail, we minimized the risk of missing nuances between synergies.

The composite first, second, third, and fourth synergies were sorted to identify best matched pairs of synergy patterns to baseline within an individual. In an iterative approach we compared the synergies in two sets by computing the similarities between the best matched pairs of weightings. The two with the highest similarity were paired, then removed from the process. The procedure was repeated until all synergies had been matched (d’Avella et al., 2003). No threshold for synergy pairing similarity was applied. Synergies were grouped by functional muscle group weightings and ordered by timings. The first synergy was weighted highest toward gluteus medius activity, second synergy weighted highest toward ankle plantarflexor muscle action, the third synergy was weighted highest toward tibialis anterior, and the highest toward semitendinosus was designated the fourth.

Similarities between synergy compositions [both muscle weightings (W) and timing co-efficient (C)] were compared by computing the cosine similarity (d’Avella et al., 2003; Muceli et al., 2010; Oliveira et al., 2014; Rimini et al., 2017). Similarity prioritizes likeness in vector shape as opposed to magnitude shift. Similarity of 1 represents perfect similarity. Following Gizzi et al. (2011) and Oliveira et al. (2014), a similarity between a pair of synergies of above 0.8 was defined as similar (Gizzi et al., 2011; Oliveira et al., 2014).

### High-Responders vs. Low-Responders

To establish a relationship between adaptability in gait and synergy complexity, we grouped individuals as high-responders and low-responders to biofeedback. High-responders were defined as those able to reach at least 5^o^ more maximal knee extension during initial contact, at least 10% increased step length, and 10% increase in ankle power generation at push off with direct biofeedback. Low-responders were defined as those able to reach no more than one of these targets. Seven participants were considered high-responders, attaining all three targets of biofeedback. Eight participants were grouped as low-responders. The remaining participants reached 2/3 of these condition targets and were excluded for this analysis.

### Statistical Analysis

To test the difference in number of synergies across feedback trials, a Wilcoxon signed-rank was used to test differences from baseline. To establish group changes in synergies across feedback trials, between-condition (baseline, step length, knee extension, and ankle power feedback) differences in tVAF_1__–__5_ were evaluated with repeated measure ANOVAs. CP and TD group outcomes for tVAF at each synergy level were compared with independent *t*-tests for baseline walking only. Differences across biofeedback trials in synergy weighting composition of the four-synergy solution were evaluated with multiple Friedman’s test; α was reduced to 0.01 to reduce Type 1 error. *Post hoc* testing was carried out to indicate significant changes from baseline. To explore differences in high-responders and low-responders, group average tVAF_1_ was compared using a Two-Way RM-ANOVA, with feedback condition and group (high-responders/low-responders) as factors. The number of synergies during baseline for each group was also compared using a Wilcoxon signed-rank test. Analyses were performed using SPSS software (IBM SPSS Statistics 23, Armonk, NY, United States) with standard α = 0.05 and Bonferroni correction applied for multiple *post hoc* comparisons.

## Results

**FIGURE 2:**
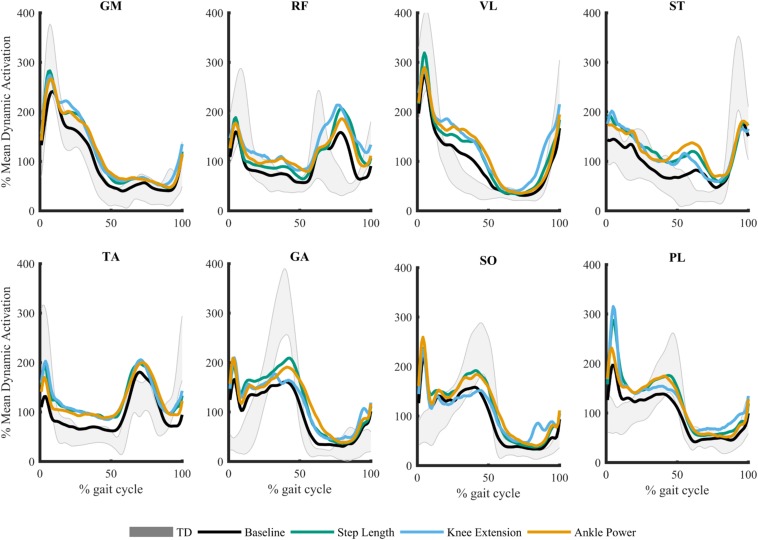
Mean muscle activation pattern across gait cycle during biofeedback trials. Gray shaded area represents average typically developing (TD) data during treadmill walking ± 1SD. Gluteus medius (GM), rectus femoris (RF), vastus lateralis (VL), semitendinosus (ST), tibialis anterior (TA), gastrocnemius medialis (GA), soleus (SO), and peroneus longus (PL).

Due to technical issues in data collection, two participants were excluded because of missing data. As such, 23 children were included in analysis (demographics presented in Table 1). In response to biofeedback, children with CP showed the capacity to adapt gait. Step length was increased 12.7 ± 11.3%, knee extension at initial contact increased by 7.4 ± 7.1^o^, and peak ankle power at push off improved 37.7 ± 36.1% (Booth et al., 2019). The grouped average, processed and normalized EMG signals for all eight muscles during the walking trials are plotted in Figure 2.

**TABLE 1:** Participant demographics in CP group (*n* = 23) and TD reference group.

**Characteristic**	**Mean ± SD or *n***	**Range**	**TD (*n* = 27)**
Age (years)	10.4 ± 3.1	6–16	10.9 ± 3.0
Height (m)	1.51 ± 0.20	1.27–1.9	1.52 ± 0.20
Body mass (kg)	41.2 ± 17.8	26.1–89	43.0 ± 12.6
Walking speed (m/s)	0.65 ± 0.18	0.35–1.1	1.05 ± 0.24
Diagnosis	CP: 21, HSP: 2		–
GMFCS	I: 11, II: 12		–
Localization	Unilateral: 8, Bilateral: 15		–
Sex	M: 16, F: 9		M: 10, F: 17

*CP, cerebral palsy; HSP, hereditary spastic paresis; GMFCS, gross motor function classification system; TD, typically developing.*

### Variance Accounted for

**FIGURE 3:**
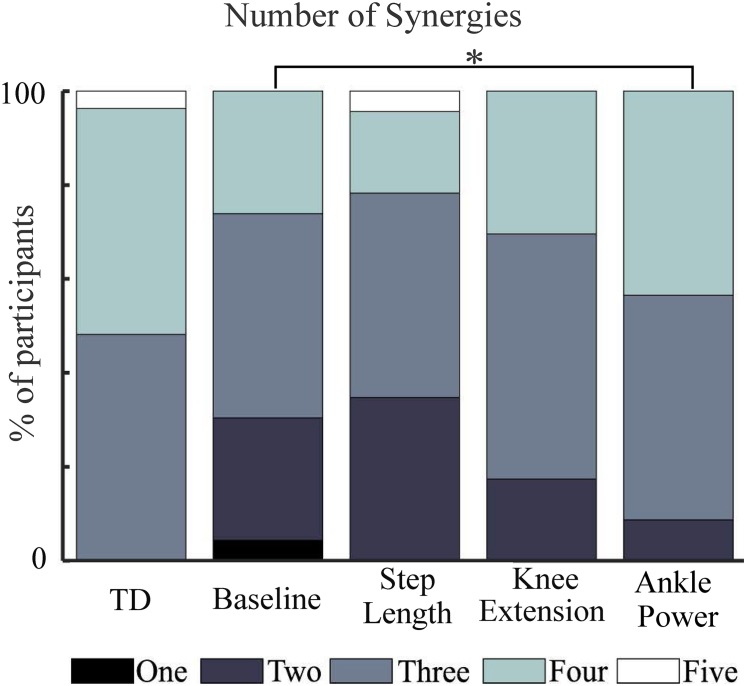
Number of synergies required to describe at least 90% of measured muscle activation across walking trials. TD, shown for reference. ^∗^ indicates significant difference to baseline (*p* = 0.008, Wilcoxon signed-rank).

A three-synergy solution described at least 90% of tVAF in 13 out of 27 TD children during baseline walking, while 13 TD children required four synergies and one child required five. In contrast, most children with CP required two or three synergies (Figure 3). During feedback trials, in children with CP, the number of synergies required to describe ≥90% of tVAF changed for some individuals. With biofeedback on ankle power, ten children required one extra synergy to describe ≥90% of tVAF, one child required two extra synergies while one required one fewer synergy; this amounted to a significant increase in the number of synergies compared to baseline (*Z* = −2.673, *p* = 0.008). No significant changes were found with biofeedback on knee extension or step length (Figure 3).

**FIGURE 4:**
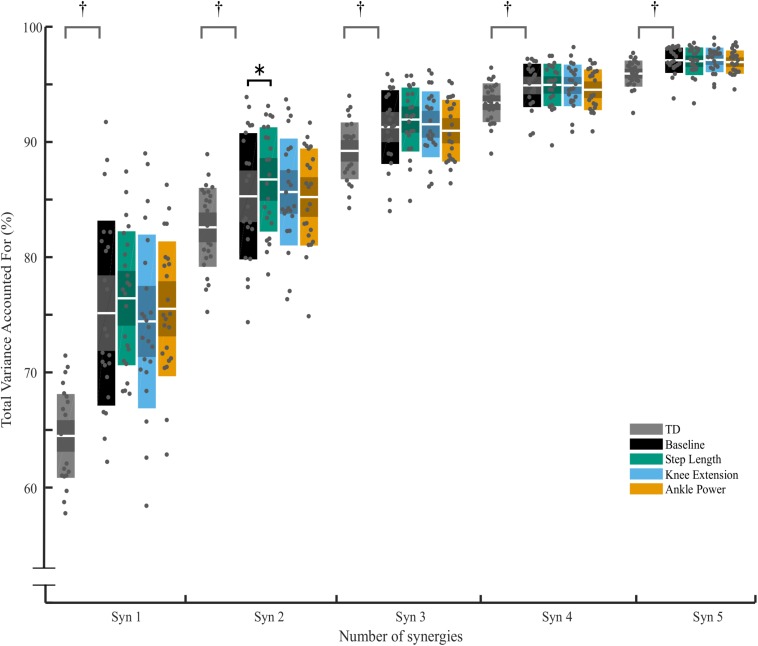
Total variance accounted for (tVAF) from one to five synergy solutions for typically developing children (TD) and for children with CP across biofeedback trials. For each cluster, individual scores are plotted with dots, white middle bar shows mean, shading shows 95% CI, while solid color represents SD. † represents significant difference between TD and CP group (*p* < 0.05, independent *t*-test). ^∗^ Significant effect of biofeedback was found in tVAF_2_ (*p* = 0.046, *post hoc* RM-ANOVA). No further significant differences between baseline and any of the biofeedback conditions were found (*p* > 0.05).

Changes in tVAF were small – for all individuals who changed by one synergy level, this was the result of a small (<5%) change in tVAF (Mean Δ: 2.2 ± 1.3%). Group average tVAF_1_ showed no difference, baseline: 0.75 ± 0.08, step length: 0.76 ± 0.05, knee extension: 0.74 ± 0.07, and ankle power 0.76 ± 0.05. No change was found in tVAF_1__,__3__,__4__,__5_ with any of the biofeedback conditions (*p* > 0.05) (Figure 4). There was a significant effect of biofeedback in tVAF_2_, with *post hoc* tests showing tVAF_2_ during step length biofeedback was significantly higher than baseline [*F*(3,66) = 2.992, *p* = 0.044]. Comparing the groups, children with CP showed less complex SMC compared to TD children as measured by tVAF_1_ (Figure 4), with lower scores for TD (0.64 ± 0.03) than for the CP group (0.75 ± 0.08) during baseline walking [*t* (48) = 6.200, *p* < 0.001; tVAF_2__–__5__,_ *p* < 0.05].

### Within-Subject Similarity

**FIGURE 5:**
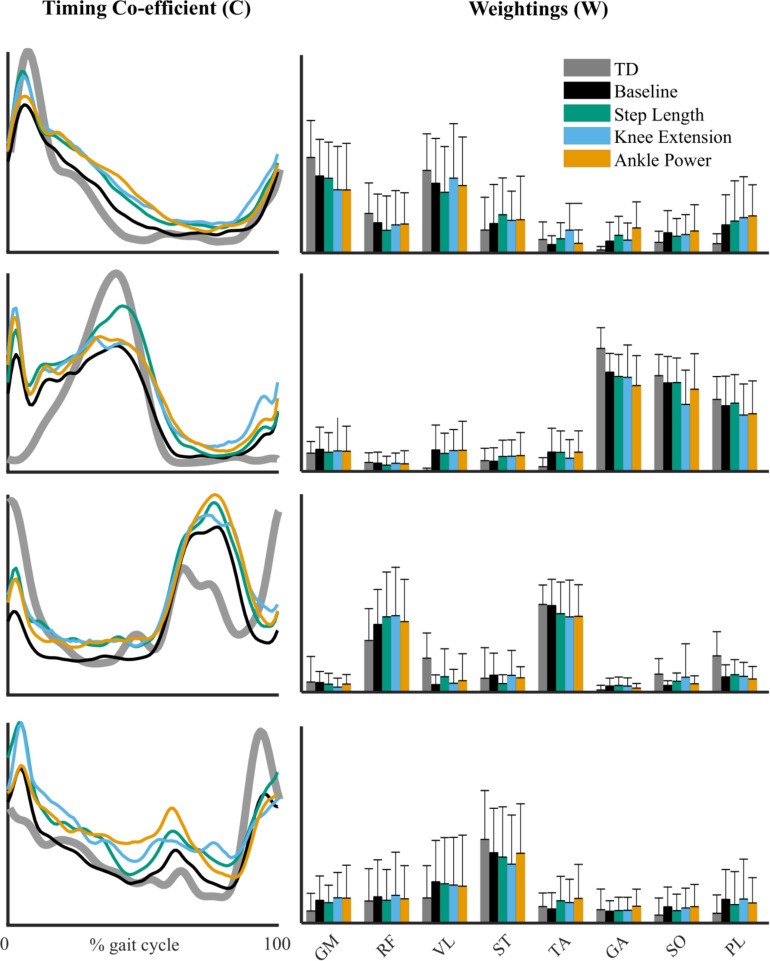
Synergy timing pattern **(left)** for the four-synergy solution. Corresponding muscle weightings **(right)** of each synergy pattern across feedback trials. Reference TD pattern shown in gray. Gluteus medius (GM), rectus femoris (RF), vastus lateralis (VL), semitendinosus (ST), tibialis anterior (TA), gastrocnemius medialis (GA), soleus (SO), and peroneus longus (PL). No significant difference in muscle weightings from baseline in *post hoc* testing (*p* > 0.01, RM-ANOVA).

**FIGURE 6:**
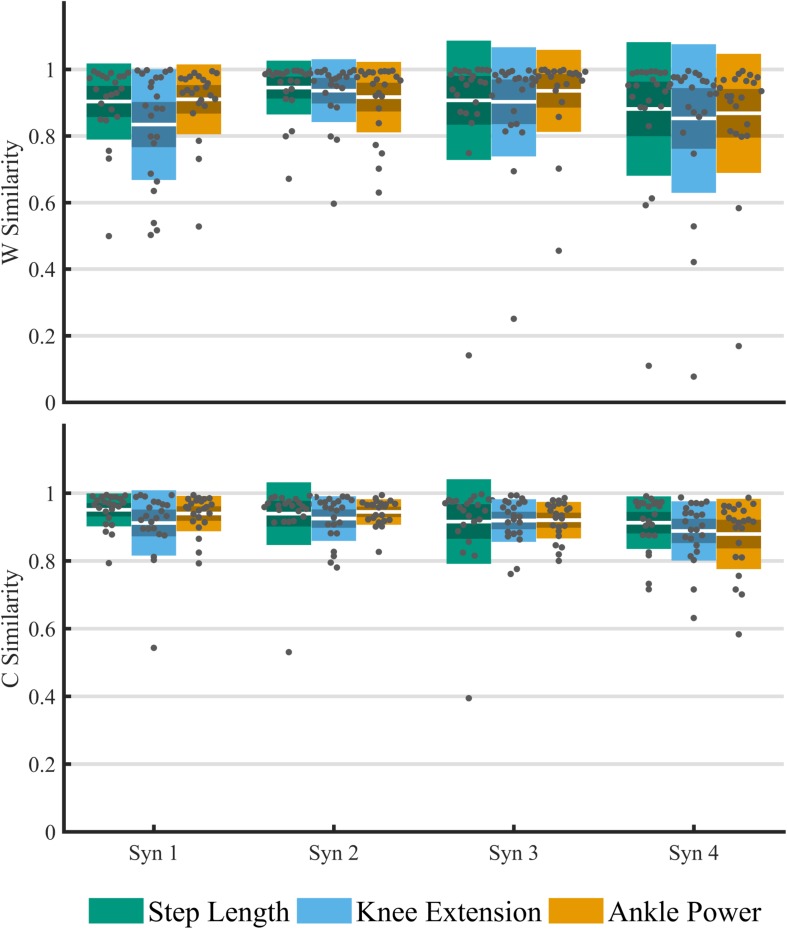
Analysis of the within-subject cosine similarity to baseline of matched paired muscle synergies for an imposed four-synergy solution across feedback trials. Similarity of weightings **(top)** and timing co-efficient **(bottom)**. For each cluster, individual results are plotted with dots, white middle bar shows mean, shading shows 95% CI, while solid color represents SD.

The structure of synergy weightings and timings between feedback trials remained relatively consistent across the group (Figure 5). There was no significant interaction effect of feedback on composition muscle weightings (*p* > 0.01). High within-subject similarities (>0.8) to baseline were found in both weighting and timing (Figure 6). Across the four-synergy solution, weightings showed similarity to baseline of 0.91 ± 0.08, 0.88 ± 0.09, and 0.90 ± 0.07 for step length, knee extension, and ankle power feedback, respectively. Synergy timings showed a similar trend, with similarities of 0.93 ± 0.06, 0.91 ± 0.06, and 0.92 ± 0.04 for step length, knee extension, and ankle power feedback, respectively. Individual responses across trials are shown in Supplementary Data Sheet 1.

### High-Responders vs. Low-Responders

The high-responders (*n* = 7) were aged 12.7 ± 2.1 years; GMFCS I = 3, II = 4; and unilateral = 1, bilateral = 6. Low-responders (*n* = 8) were aged 9.1 ± 2.7 years; GMFCS I = 4, II = 4; and unilateral = 5, bilateral = 3. The responders group showed an average tVAF_1_ score at baseline of 0.74 ± 0.08 compared to 0.78 ± 0.07 for the non-responders (*p* = 0.311, independent *t*-test). The results of the two-way mixed ANOVA showed that there was no significant interaction effect between high-responders/low-responders and biofeedback condition on tVAF_1_ [*F*(3,39) = 2.126, *p* = 0.130, η_p_^2^ = 0.141]. No difference was found in the number of synergies required in the high-responders group (3.1 ± 0.9) and low-responder group (2.6 ± 0.7) during baseline walking and across all biofeedback trials (*p* = 0.370). Grouped synergy composition of high-responders and low-responders and shown in Supplementary Data Sheet 2.

## Discussion

The goal of this study was to explore the underlying SMC in children with CP associated with changes in gait in response to real-time biofeedback during treadmill walking. Children with CP showed adaptability in gait to reach the targets of biofeedback, reaching significant and meaningful improvements in gait kinematics, kinetics, and spatiotemporal parameters. When considering SMC, we found contradictory results with a small yet significant change in tVAF and number of synergies required to describe SMC in response to biofeedback. Within-subject similarity of weightings and timing for an imposed four-synergy solution were high across the feedback trials. These results suggest that while gait may be adapted in an immediate response to biofeedback, it is questionable if this involves an adaptation of SMC.

In this study we found subtle changes in synergies between comfortable walking and adapted walking with biofeedback. We observed changes when considering the number of synergies required to reach a threshold of at least 90% tVAF (Clark et al., 2010; Dominici et al., 2011; Steele et al., 2015). Eleven participants required more synergies to describe muscle activity during walking with ankle power feedback than during baseline walking. Both individual and group differences in tVAF, however, were small (Figure 4). For all individuals who changed by one synergy level, this was the result of a small (<5%) change in tVAF (Mean Δ: 2.2 ± 1.3%). We found a change in group tVAF_2_, with values significantly higher during step length biofeedback than during baseline, which could indicate slightly adaptable SMC. However, absolute difference in mean tVAF_2_ between baseline and step length trials was small, less than 2% tVAF. Further investigation is required to establish meaningful change at the level of an individual; therefore, it may be questioned if these small changes are clinically relevant. The limited change in synergy structure is also reflected in the EMG profiles; while amplitude is adapted, the activation pattern shows high similarity to baseline walking (>0.93 cosine similarity). Moreover, the structure of synergistic action showed high consistency across trials. When investigating the imposed four-synergy solution, we observed high within-subject similarity (>0.8) of both weighting and timing of synergies. This result ties with recent findings that – following intervention in children with CP and significant changes in gait – while changes in the number of synergies are found, there is high similarity (Shuman et al., 2019). Further to this, TD children show consistent muscle synergies when walking at different speeds and slopes (Rozumalski et al., 2017). This provides further evidence toward the theory that synergies are neural in nature and are consistent over similar functional tasks. Similarity of synergies as found in our study is comparable to reported studies (Routson et al., 2014; Rimini et al., 2017; Rozumalski et al., 2017; Shuman et al., 2019).

The alternative explanation for individual variation in weightings and timings is that biofeedback-driven gait adaptations are in fact resulting in subtly adapted SMC, reflected in slight changes in synergy composition. It should be noted, however, that one of the limitations of using cosine similarity is that the similarity due to chance is high (∼0.75). Therefore, changes under this value may reflect significant adaptation. However, given the minor changes in tVAF and high overall similarity, we cannot convincingly support this from our findings.

Contrary to expectations, we could not establish a trend toward the use of tVAF as a predictor of adaptability in gait. Similar to previous findings, we showed that ambulatory children with CP show a higher tVAF at each synergy level when compared to TD children (Steele et al., 2015; Cappellini et al., 2016, 2018; Goudriaan et al., 2018). This is further evidence that synergy analysis may relate to functional motor impairments, with tVAF_1_ indicative for the complexity of SMC. If individuals with lower tVAF_1_ do have a capacity for more complex SMC, it may be expected that they would also have greater ability to adapt gait in response to biofeedback by a more refined recruitment of muscles. Our results cannot confirm this hypothesis, as no difference was found between high-responders and low-responders to the challenges of biofeedback. However, we were limited in this analysis by a small subsample size. In addition, the functional differences between the groups were small. All participants were ambulatory children, and all were able to adapt gait to some extent in response to biofeedback. Further exploration of this concept with larger and more distinct populations is required to establish the use of tVAF_1_ as a proxy measure of the capacity of an individuals’ gait adaptability.

There are limitations in inferring SMC from the presented method of synergy analysis. The use of synergies as a measure of SMC is not yet fully understood, and there is a lack of general consensus (Tresch and Jarc, 2009). One critique is that muscle synergies reflect a functional task constraint rather than neural control strategies (De Groote et al., 2014). In our study, the functional task of walking was similar between conditions. While gait changes in kinematics were considerable, the individuals were carrying out a highly similar functional task: walking. For example, in animal models there is evidence of shared synergies even between differing functional tasks such as walking, swimming and jumping (d’Avella et al., 2003). The targets of biofeedback were not chosen as those that may have the largest effect on synergy composition, more so toward clinical improvements in walking. There is evidence to suggest that plantarflexor activity may be particularly important for synergy composition and walking ability (Routson et al., 2013; Goudriaan et al., 2018). While ankle power may be considered a proxy for plantarflexor activation, perhaps targeting this specifically with biofeedback will yield more significant changes.

The presented method of synergy analysis may not be sensitive enough to quantify these adaptations in gait. Synergy analysis is influenced by the number and selection of specific muscles (Steele et al., 2013). To reveal the complexity of synergies, concatenating at least 20 steps is recommended (Oliveira et al., 2014). It may be of interest to consider strides individually to investigate variability. This study does not report step-to-step variability; tVAF can be reduced by increasing variability, and this may have been induced by the novel biofeedback condition. This potentially explains some of the changes we see in tVAF. By concatenating a large number of strides, we hoped to reduce this effect. Children with CP exhibit greater stride-to-stride variability than TD children, and this may relate to walking impairment (Kim et al., 2018). With further practice it may be that timing and variation can be improved, resulting in functional improvements. Synergies are also sensitive to data processing of EMG (Shuman et al., 2017). By implementing a standard method of EMG processing, we can expect to minimize these discrepancies. Eight muscles from one leg were used in the present study. Gait, however, is a whole-body movement and many muscles across the body contribute to overall function. An important limitation of this study is that biofeedback was provided on only the most affected side. In addition, EMG analysis is reported only on this side. As such, we cannot quantify the resultant effect on the opposite leg. It may be that additional adaptations in muscle activation are being made on this leg. Future studies would benefit from bilateral EMG analysis to provide a comprehensive overview of SMC. By measuring a limited number of muscles, it may be difficult to fully establish the underlying SMC. Activation of small or deep muscles that were not measured in this present study may have important influence. It is likely that synergies provide the building block for functional movement, but these may be supplemented with refined control to achieve specific modifications in movement. In our measured EMG activity, a peak around initial contact is evident. It is expected that this is the result of hyper-reflexive reaction to muscle stretch following initial contact. Many of the subjects reported spasticity of the calf muscles and walked in a toe-gait pattern. This activity may influence the extrapolation of synergies.

Our study population consisted of a range of ages, functional ability, and diagnosis. These factors influence the composition of synergies and may contribute to the lack of change identified at a group level. While adults with HSP have been shown to display impaired locomotor co-ordination (Martino et al., 2019), further research is required to directly compare CP and HSP pediatric populations. The lack of clear changes in muscle synergies may have implications for rehabilitation of neurological impairment. Synergies have been shown to be related to functional impairment (Steele et al., 2015), with more impaired individuals showing reduced effectiveness of interventions across multiple centers (Shuman et al., 2018). Given that synergies appear to be relatively consistent, it may cast doubt on the ability of individuals with neurological impairment to adapt and improve motor tasks such as walking. Nevertheless, gait training has been shown to be effective in improving walking function in individuals with impaired SMC, such as CP (Booth et al., 2018) and post-stroke (Mehrholz et al., 2017). Synergies mature over the years in early development (Dominici et al., 2011) and may be associated with developmental re-structuring of the spinal cord (Ivanenko et al., 2013). Perhaps it should not be expected that synergies are altered in a short-term response to changes in gait as done in this study. Such gait adaptation may require a prolonged period of practice before it is innately learned and recruited. In a 12-week gait training intervention for individuals post-stroke, synergy module timings were found to be somewhat adapted, alongside improved walking performance (Routson et al., 2013). To the authors’ knowledge there are no studies looking at changes in synergies associated with prolonged gait training interventions in individuals with CP. It may be that gait training with biofeedback, initiated at an early age, would be most effective in developing and learning improved SMC. With direct biofeedback, children with CP are able to alter muscle activations during gait (Bolek, 2003; Dursun et al., 2004). In combination with our results, these studies highlight the potential adaptability of gait in children with CP. Therefore, future research should investigate potential adaptation of SMC after a prolonged period of gait training with the addition of biofeedback.

## Conclusion

Children with CP can selectively adapt and improve aspects of gait when challenged with biofeedback. Subtle changes in synergy patterns and tVAF were identified; however, it is unclear if these changes are clinically important. Within-subject similarity of synergies was high across the biofeedback trials. These results suggest that while gait may be adapted in an immediate response, SMC as quantified by synergy analysis is perhaps more rigidly impaired in CP. Adaptations may be limited in the short term, and further investigation is essential to establish if long-term training using biofeedback leads to adapted SMC.

## Ethics Statement

This study was carried out in accordance with the recommendations of the Medical ethics committee of the VUMC with written informed consent from all subjects. All subjects gave written informed consent in accordance with the Declaration of Helsinki.

## Author Contributions

ATB, MK, JH, and AIB contributed to the conception and design of the study. ATB carried out the data collection, analysis, and drafting of the manuscript. ND, MK, JH, and AIB contributed to the intellectual concepts and analysis. All authors contributed to the manuscript revisions, read, and approved the submitted version.

## Conflict of Interest

ATB is employed by the Motek Medical BV in a position fully funded by the PACE ITN, under the European Union’s Horizon 2020 Research and Innovation program, Marie Skłodowska-Curie grant agreement number 642961. All research direction and integrity are supervised by the Amsterdam UMC. The remaining authors declare that the research was conducted in the absence of any commercial or financial relationships that could be construed as a potential conflict of interest.

## Abbreviations

CP: cerebral palsy
EMG: electromyography
GMFCS: gross motor function classification system
NNMF: non-negative matrix factorization
SMC: selective motor control
tVAF: total variance accounted for
TD: typically developing.

## References

[B1] Baram Y. Lenger R. (2012). Gait Improvement in patients with Cerebral Palsy by visual and auditory feedback. *Neuromodulation* 15 48–52. 10.1111/j.1525-1403.2011.00412.x 22151772

[B2] Bolek J. E. (2003). A preliminary study of modification of gait in real-time using surface electromyography. *Appl. Psychophysiol. Biofeedback* 28 129–138. 10.1023/A:1023810608949 12827991

[B3] Booth A. T. Buizer A. I. Harlaar J. Steenbrink F. van der Krogt M. M. (2019). Immediate effects of immersive biofeedback on gait in children with Cerebral Palsy. *Arch. Phys. Med. Rehabil.* 100 598–605. 10.1016/j.apmr.2018.10.013 30447196

[B4] Booth A. T. C. Buizer A. I. Meyns P. Oude Lansink I. L. B. Steenbrink F. van der Krogt M. M. (2018). The efficacy of functional gait training in children and young adults with cerebral palsy: a systematic review and meta-analysis. *Dev. Med. Child Neurol.* 60 866–883. 10.1111/dmcn.13708 29512110

[B5] Cappellini G. Ivanenko Y. P. Martino G. Maclellan M. J. Sacco A. Morelli D. (2016). Immature spinal locomotor output in children with Cerebral Palsy. *Front. Physiol.* 7:478. 10.3389/fphys.2016.00478 27826251 PMC5078720

[B6] Cappellini G. Sylos-Labini F. MacLellan M. J. Sacco A. Morelli D. Lacquaniti F. (2018). Backward walking highlights gait asymmetries in children with cerebral palsy. *J. Neurophysiol.* 119 1153–1165. 10.1152/jn.00679.2017 29357466

[B7] Chruscikowski E. Fry N. R. D. Noble J. J. Gough M. Shortland A. P. (2017). Selective motor control correlates with gait abnormality in children with cerebral palsy. *Gait Posture* 52 107–109. 10.1016/j.gaitpost.2016.11.031 27889619

[B8] Clark D. J. Ting L. H. Zajac F. E. Neptune R. R. Kautz S. A. (2010). Merging of healthy motor modules predicts reduced locomotor performance and muscle coordination complexity post-stroke. *J. Neurophysiol.* 103 844–857. 10.1152/jn.00825.2009 20007501 PMC2822696

[B9] d’Avella A. Saltiel P. Bizzi E. (2003). Combinations of muscle synergies in the construction of a natural motor behavior. *Nat. Neurosci.* 6 300–308. 10.1038/nn1010 12563264

[B10] De Groote F. Jonkers I. Duysens J. (2014). Task constraints and minimization of muscle effort result in a small number of muscle synergies during gait. *Front. Comput. Neurosci.* 8:115. 10.3389/fncom.2014.00115 25278871 PMC4167006

[B11] Dominici N. Ivanenko Y. P. Cappellini G. D’Avella A. Mondi V. Cicchese M. (2011). Locomotor primitives in newborn babies and their development. *Science* 334 997–999. 10.1126/science.1210617 22096202

[B12] Dursun E. Dursun N. Alican D. (2004). Effects of biofeedback treatment on gait in children with cerebral palsy. *Disabil. Rehabil.* 26 116–120. 10.1080/09638280310001629679 14668149

[B13] Falisse A. Van Rossom S. Gijsbers J. Steenbrink F. van Basten B. J. H. Jonkers I. (2018). OpenSim versus human body model: a comparison study for the lower limbs during gait. *J. Appl. Biomech.* 10.1123/jab.2017-0156 [Epub ahead of print]. 29809082

[B14] Fowler E. G. Staudt L. A. Greenberg M. B. (2010). Lower-extremity selective voluntary motor control in patients with spastic cerebral palsy: increased distal motor impairment. *Dev. Med. Child Neurol.* 52 264–269. 10.1111/j.1469-8749.2009.03586.x 20089048

[B15] Fowler E. G. Staudt L. A. Greenberg M. B. Oppenheim W. L. (2009). Selective control assessment of the lower extremity (SCALE): development, validation, and interrater reliability of a clinical tool for patients with cerebral palsy. *Dev. Med. Child Neurol.* 51 607–614. 10.1111/j.1469-8749.2008.03186.x 19220390

[B16] Gizzi L. Nielsen J. F. Felici F. Ivanenko Y. P. Farina D. (2011). Impulses of activation but not motor modules are preserved in the locomotion of subacute stroke patients. *J. Neurophysiol.* 106 202–210. 10.1152/jn.00727.2010 21511705

[B17] Goudriaan M. Shuman B. R. Steele K. M. Van den Hauwe M. Goemans N. Molenaers G. (2018). Non-neural muscle weakness has limited influence on complexity of motor control during gait. *Front. Hum. Neurosci.* 12:5. 10.3389/fnhum.2018.00005 29445330 PMC5797794

[B18] Hermens H. J. Freriks B. Disselhorst-Klug C. Rau G. (2000). Development of recommendations for SEMG sensors and sensor placement procedures. *J. Electromyogr. Kinesiol.* 10 361–374. 10.1016/S1050-6411(00)00027-4 11018445

[B19] Ivanenko Y. P. Dominici N. Cappellini G. Di Paolo A. Giannini C. Poppele R. E. (2013). Changes in the spinal segmental motor output for stepping during development from infant to adult. *J. Neurosci.* 33 3025–3036. 10.1523/JNEUROSCI.2722-12.2013 23407959 PMC6619203

[B20] Kim Y. Bulea T. C. Damiano D. L. (2018). Children With Cerebral Palsy have greater stride-to-stride variability of muscle synergies during gait than typically developing children: implications for motor control complexity. *Neurorehabil. Neural Repair* 32 834–844. 10.1177/1545968318796333 30223739 PMC7271466

[B21] Martino G. Ivanenko Y. Serrao M. Ranavolo A. Draicchio F. Casali C. (2019). Locomotor coordination in patients with Hereditary Spastic Paraplegia. *J. Electromyogr. Kinesiol.* 45 61–69. 10.1016/j.jelekin.2019.02.006 30836301

[B22] Mehrholz J. Pohl M. Elsner B. (2017). Treadmill training and body weight support for walking after stroke. *Cochrane Database Syst. Rev.* CD002840. 10.1002/14651858.CD002840.pub4 28815562 PMC6483714

[B23] Muceli S. Boye A. T. d’Avella A. Farina D. (2010). Identifying representative synergy matrices for describing muscular activation patterns during multidirectional reaching in the horizontal plane. *J. Neurophysiol.* 103 1532–1542. 10.1152/jn.00559.2009 20071634

[B24] Oliveira A. S. Gizzi L. Farina D. Kersting U. G. (2014). Motor modules of human locomotion: influence of EMG averaging, concatenation, and number of step cycles. *Front. Hum. Neurosci.* 8:335. 10.3389/fnhum.2014.00335 24904375 PMC4033063

[B25] Palisano R. Rosenbaum P. Bartlett D. Livingston M. Walter S. Russell D. (1997). Gross motor function classification system. *Dev. Med. Child Neurol.* 39 214–223. 9183258 10.1111/j.1469-8749.1997.tb07414.x

[B26] Rimini D. Agostini V. Knaflitz M. (2017). Intra-subject consistency during locomotion: similarity in shared and subject-specific muscle synergies. *Front. Hum. Neurosci.* 11:586. 10.3389/fnhum.2017.00586 29255410 PMC5723022

[B27] Routson R. L. Clark D. J. Bowden M. G. Kautz S. A. Neptune R. R. (2013). The influence of locomotor rehabilitation on module quality and post-stroke hemiparetic walking performance. *Gait Posture* 38 511–517. 10.1016/j.gaitpost.2013.01.020 23489952 PMC3687005

[B28] Routson R. L. Kautz S. A. Neptune R. R. (2014). Modular organization across changing task demands in healthy and poststroke gait. *Physiol. Rep.* 2 1–14. 10.14814/phy2.12055 24963035 PMC4208640

[B29] Rozumalski A. Steele K. M. Schwartz M. H. (2017). Muscle synergies are similar when typically developing children walk on a treadmill at different speeds and slopes. *J. Biomech.* 64 112–119. 10.1016/j.jbiomech.2017.09.002 28943157

[B30] Sanger T. D. Chen D. Delgado M. R. Gaebler-Spira D. Hallett M. Mink J. W. (2006). Definition and classification of negative motor signs in childhood. *Pediatrics* 118 2159–2167. 10.1542/peds.2005-3016 17079590

[B31] Schwartz M. H. Rozumalski A. Steele K. M. (2016). Dynamic motor control is associated with treatment outcomes for children with cerebral palsy. *Dev. Med. Child Neurol.* 58 1139–1145. 10.1111/dmcn.13126 27097830 PMC8912927

[B32] Shuman B. R. Goudriaan M. Desloovere K. Schwartz M. H. Steele K. M. (2018). Associations between muscle synergies and treatment outcomes in cerebral palsy are robust across clinical centers. *Arch. Phys. Med. Rehabil.* 99 2175–2182. 10.1016/j.apmr.2018.03.006 29649451 PMC6179956

[B33] Shuman B. R. Goudriaan M. Desloovere K. Schwartz M. H. Steele K. M. (2019). Muscle synergies demonstrate only minimal changes after treatment in cerebral palsy. *J. Neuroeng. Rehabil.* 161:46. 10.1186/s12984-019-0502-3 30925882 PMC6441188

[B34] Shuman B. R. Schwartz M. H. Steele K. M. (2017). Electromyography data processing impacts muscle synergies during gait for unimpaired children and children with Cerebral Palsy. *Front. Comput. Neurosci.* 11:50. 10.3389/fncom.2017.00050 28634449 PMC5460588

[B35] Steele K. M. Rozumalski A. Schwartz M. H. (2015). Muscle synergies and complexity of neuromuscular control during gait in cerebral palsy. *Dev. Med. Child Neurol.* 57 1176–1182. 10.1111/dmcn.12826 26084733 PMC4683117

[B36] Steele K. M. Tresch M. C. Perreault E. J. (2013). The number and choice of muscles impact the results of muscle synergy analyses. *Front. Comput. Neurosci.* 7:105. 10.3389/fncom.2013.00105 23964232 PMC3737463

[B37] Tang L. Li F. Cao S. Zhang X. Wu D. Chen X. (2015). Muscle synergy analysis in children with cerebral palsy. *J. Neural Eng.* 12:046017. 10.1088/1741-2560/12/4/046017 26061115

[B38] Ting L. H. Macpherson J. M. (2005). A limited set of muscle synergies for force control during a postural task. *J. Neurophysiol.* 93 609–613. 10.1152/jn.00681.2004 15342720

[B39] Tresch M. C. Jarc A. (2009). The case for and against muscle synergies. *Curr. Opin. Neurobiol.* 19 601–607. 10.1016/j.conb.2009.09.002 19828310 PMC2818278

[B40] Tresch M. C. Saltiel P. D’Avella A. Bizzi E. (2002). Coordination and localization in spinal motor systems. *Brain Res. Brain Res. Rev.* 40 66–79. 10.1016/s0165-0173(02)00189-3 12589907

[B41] van den Bogert A. J. Geijtenbeek T. Even-Zohar O. Steenbrink F. Hardin E. C. (2013). A real-time system for biomechanical analysis of human movement and muscle function. *Med. Biol. Eng. Comput.* 51 1069–1077. 10.1007/s11517-013-1076-z 23884905 PMC3751375

[B42] van Gelder L. Booth A. T. C. van de Port I. Buizer A. I. Harlaar J. van der Krogt M. M. (2017). Real-time feedback to improve gait in children with cerebral palsy. *Gait Posture* 52 76–82. 10.1016/j.gaitpost.2016.11.021 27883988

[B43] Zeni J. A. Richards J. G. Higginson J. S. (2008). Two simple methods for determining gait events during treadmill and overground walking using kinematic data. *Gait Posture* 27 710–714. 10.1016/j.gaitpost.2007.07.007 17723303 PMC2384115

