# Supplementary material for: Muscle Synergies in Response to Biofeedback-Driven Gait Adaptations in Children With Cerebral Palsy
Source: Front Physiol. 2019 Sep 27;10:1208. doi: 10.3389/fphys.2019.01208 (PMC6776587; doi:10.3389/fphys.2019.01208)
Supplement: Supplementary file 1 [file Data_Sheet_1.pdf]

Supplementary 1. Individual participants plots for synergy timing pattern (left) for the four-synergy solution. Corresponding muscle weightings (right) of each synergy pattern across feedback trials. Gluteus medius (GM), rectus femoris (RF), vastus lateralis (VL), semitendinosus (ST), tibialis anterior (TA), gastrocnemius medialis (GA), soleus (SO), and peroneus longus (PL).

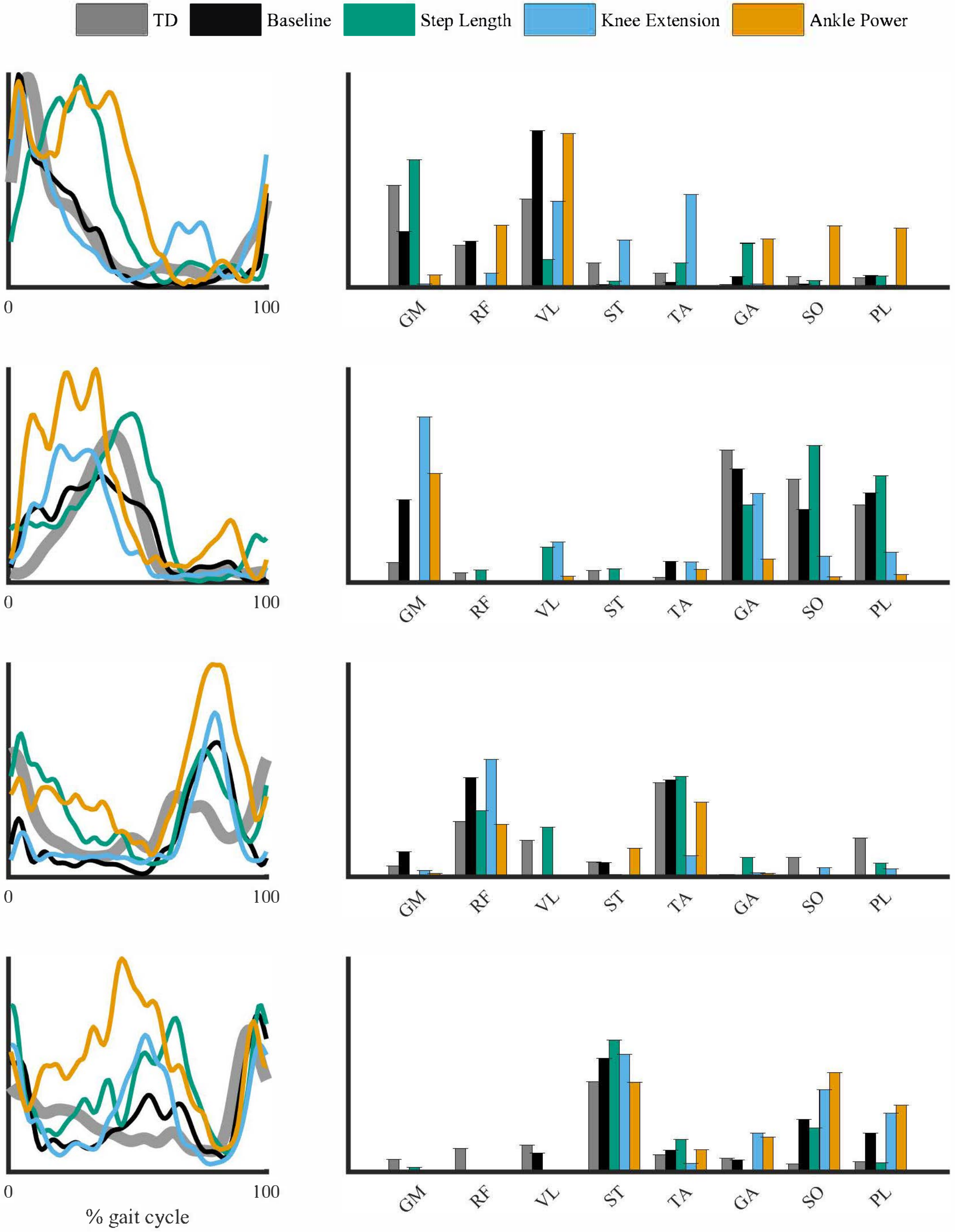

***High-responder to biofeedback**

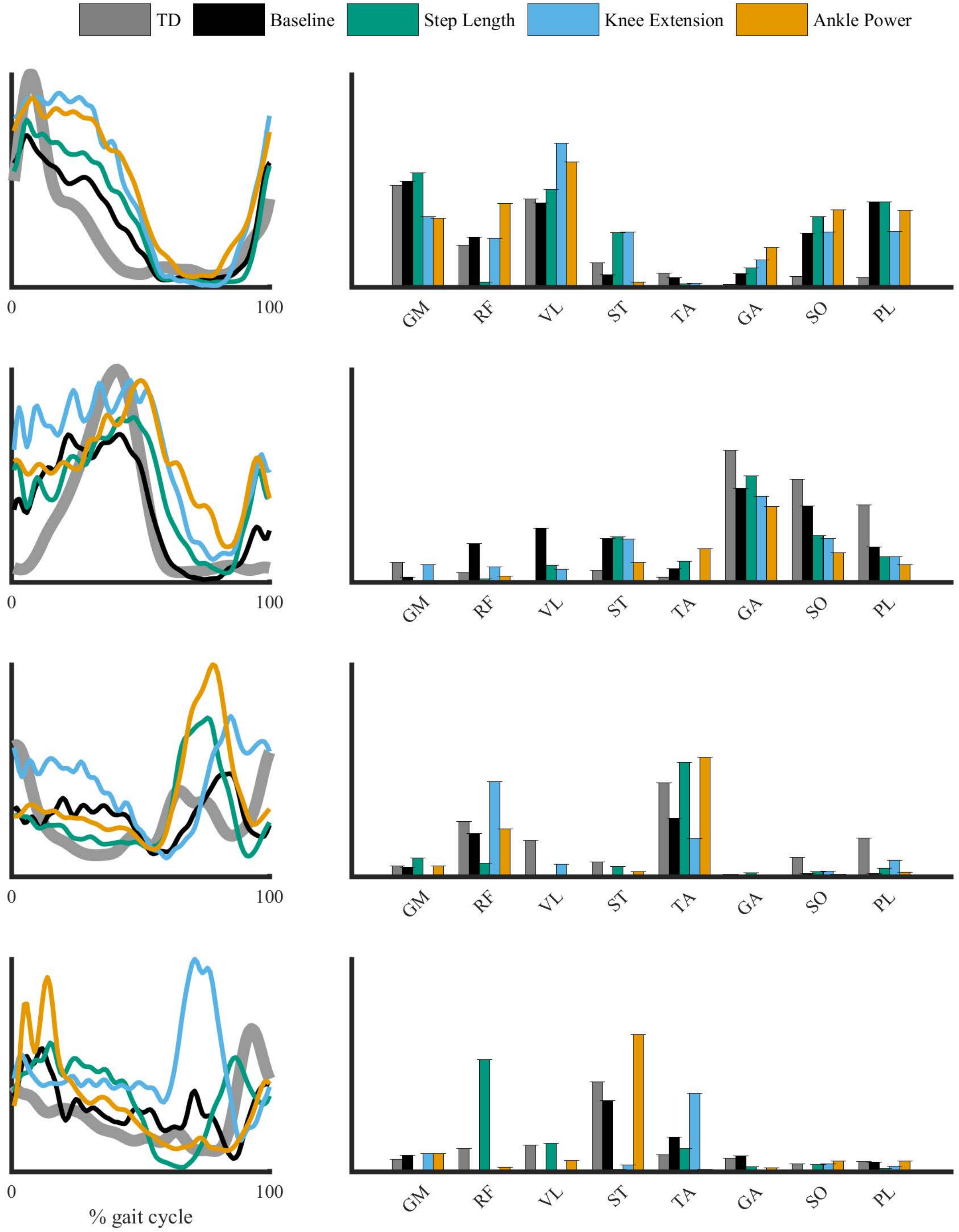

TD
Baseline
Step Length
Knee Extension
Ankle Power
0
100
GM
RF
VL
ST
TA
GA
SO
PL
% gait cycle

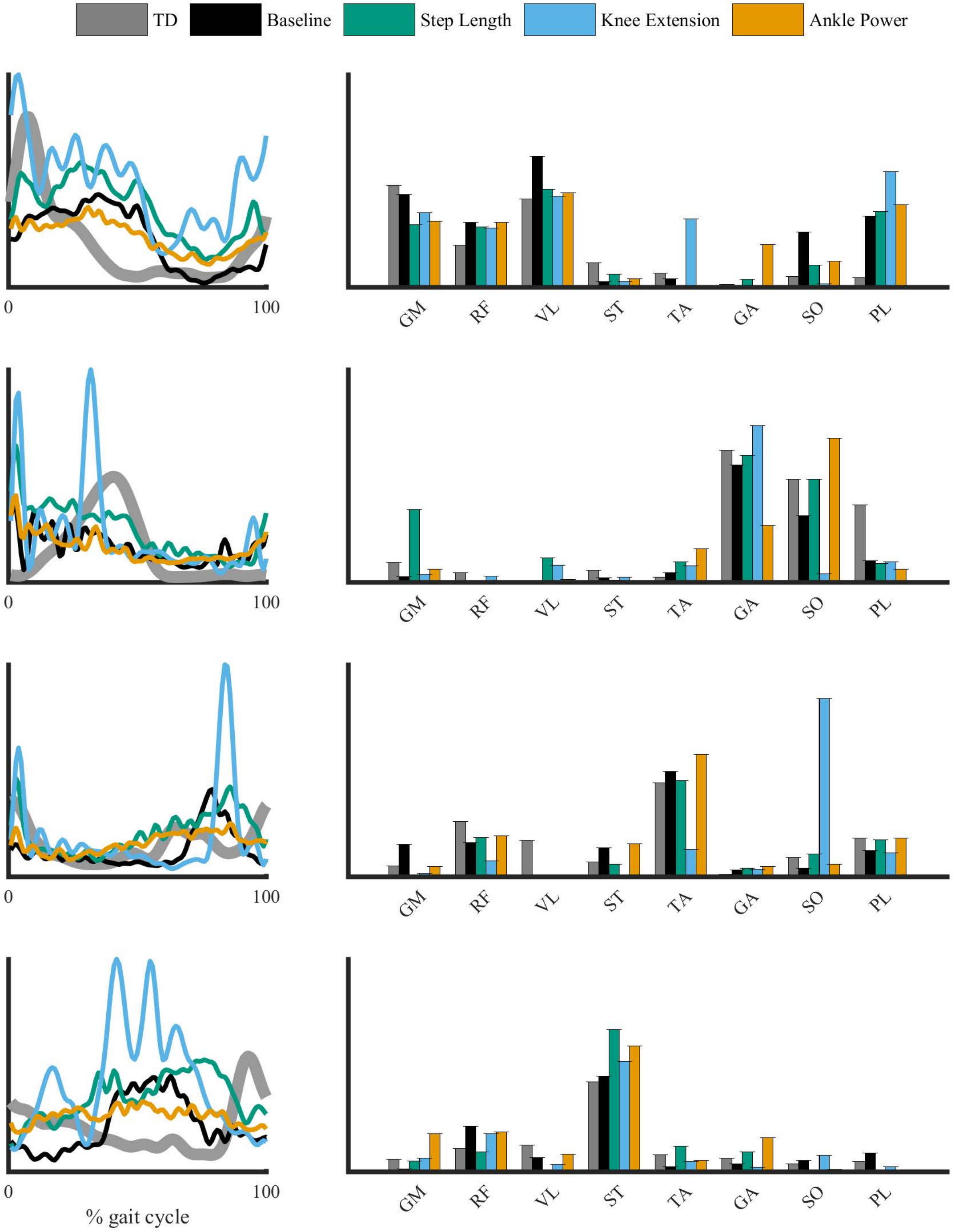
TD
Baseline
Step Length
Knee Extension
Ankle Power
0
100
GM
RF
VL
ST
TA
GA
SO
PL
% gait cycle

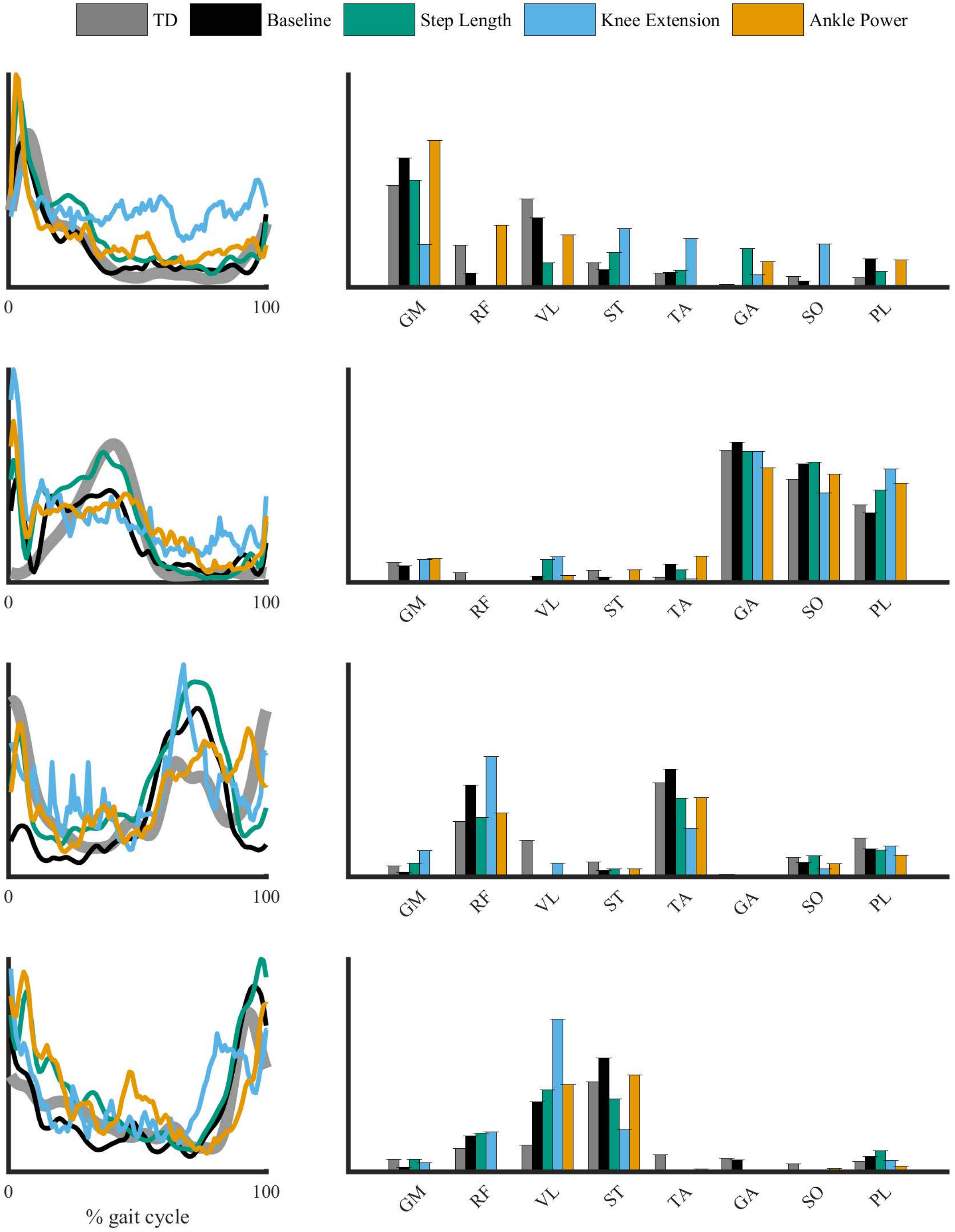

TD
Baseline
Step Length
Knee Extension
Ankle Power
0
100
GM
RF
VL
ST
TA
GA
SO
PL
% gait cycle

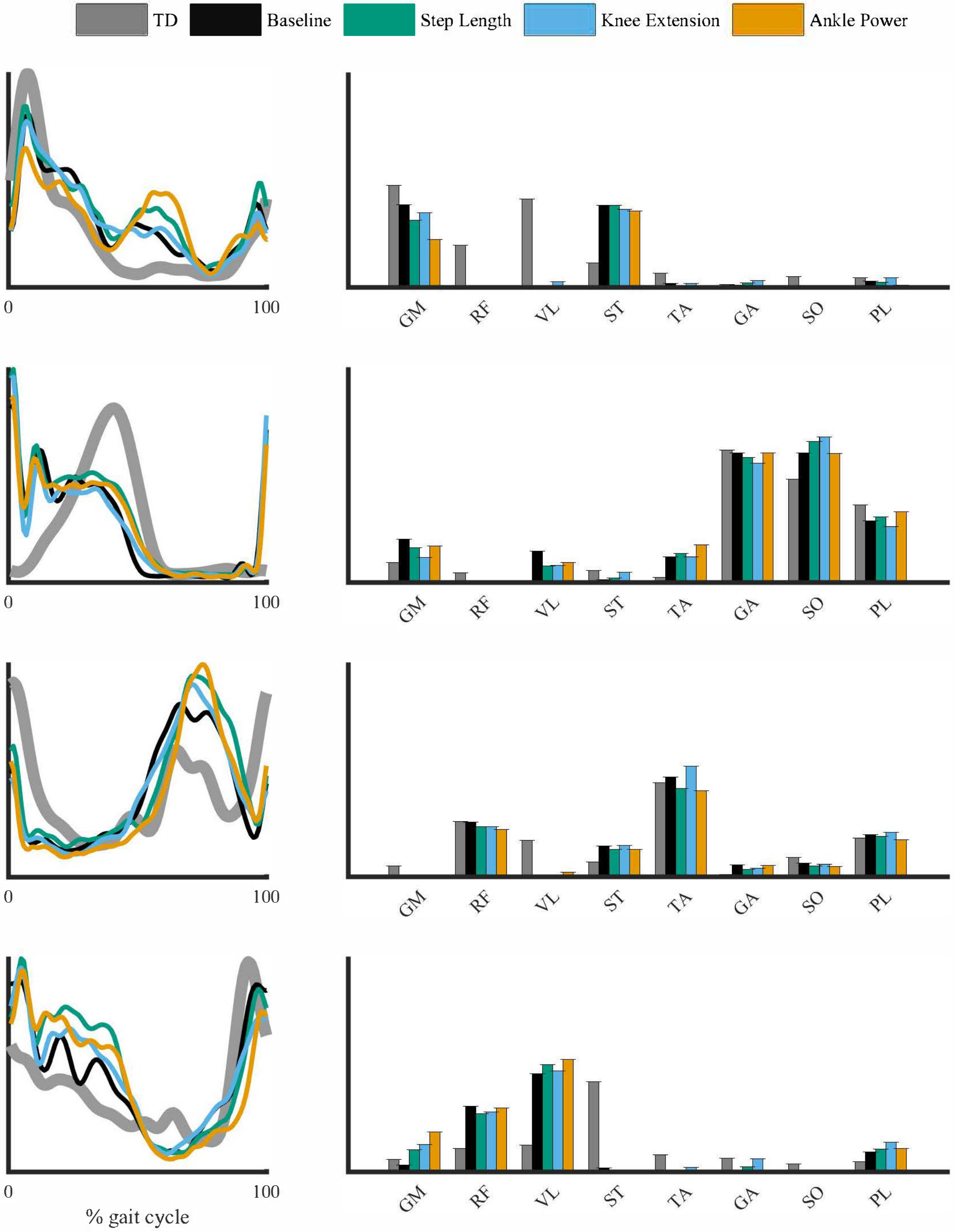

***Low-responder to biofeedback**

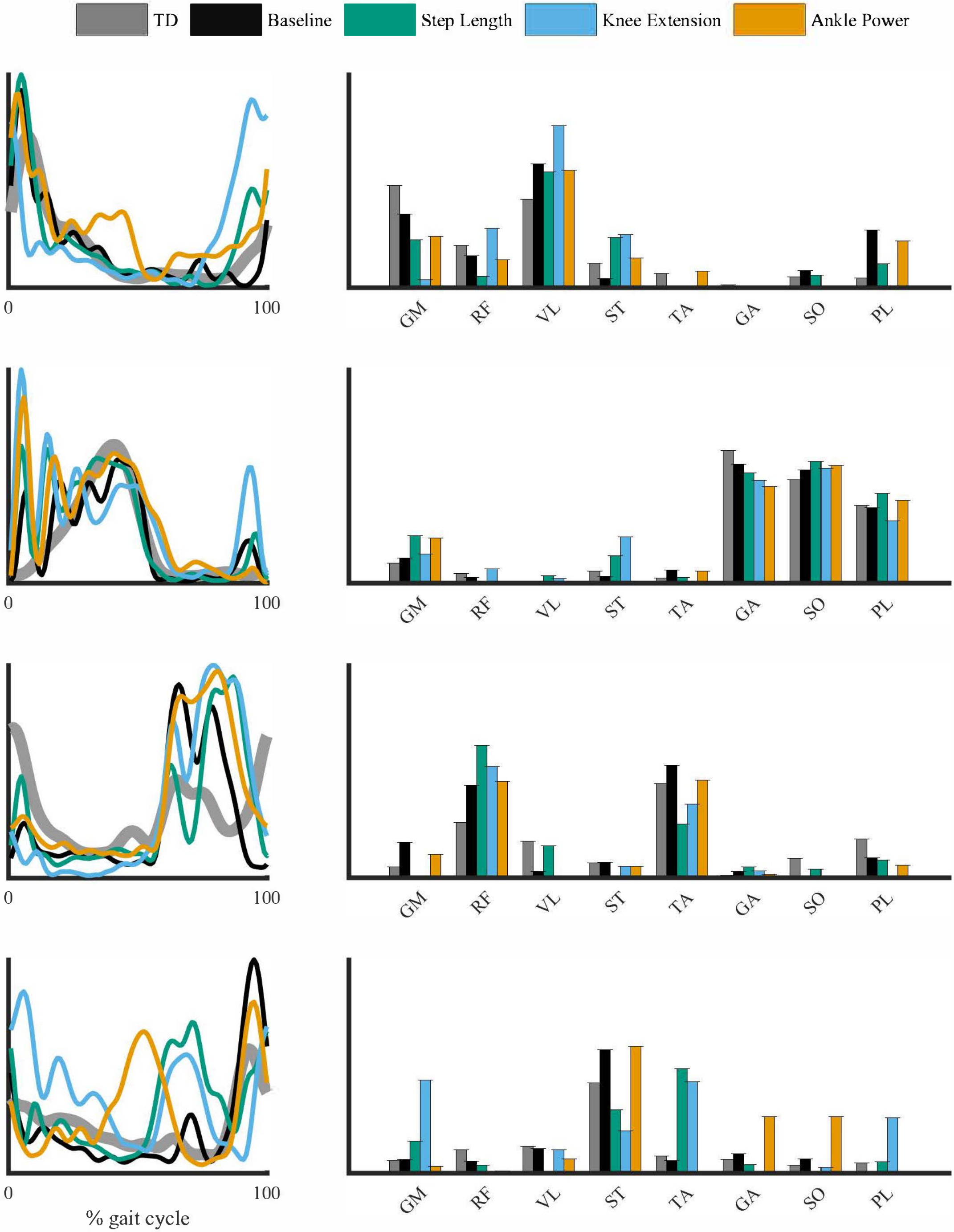

*High-responder to biofeedback

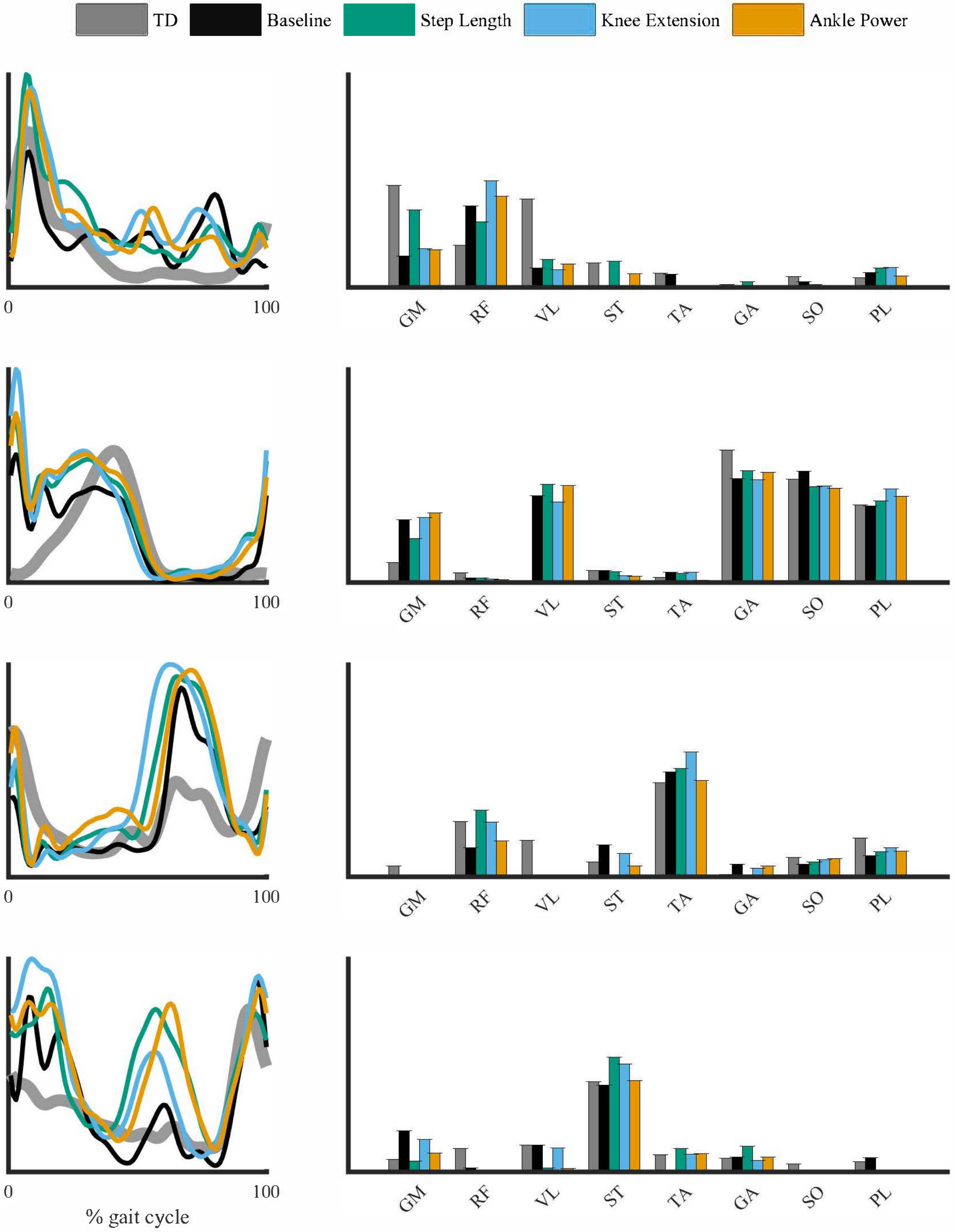

*Low-responder to biofeedback

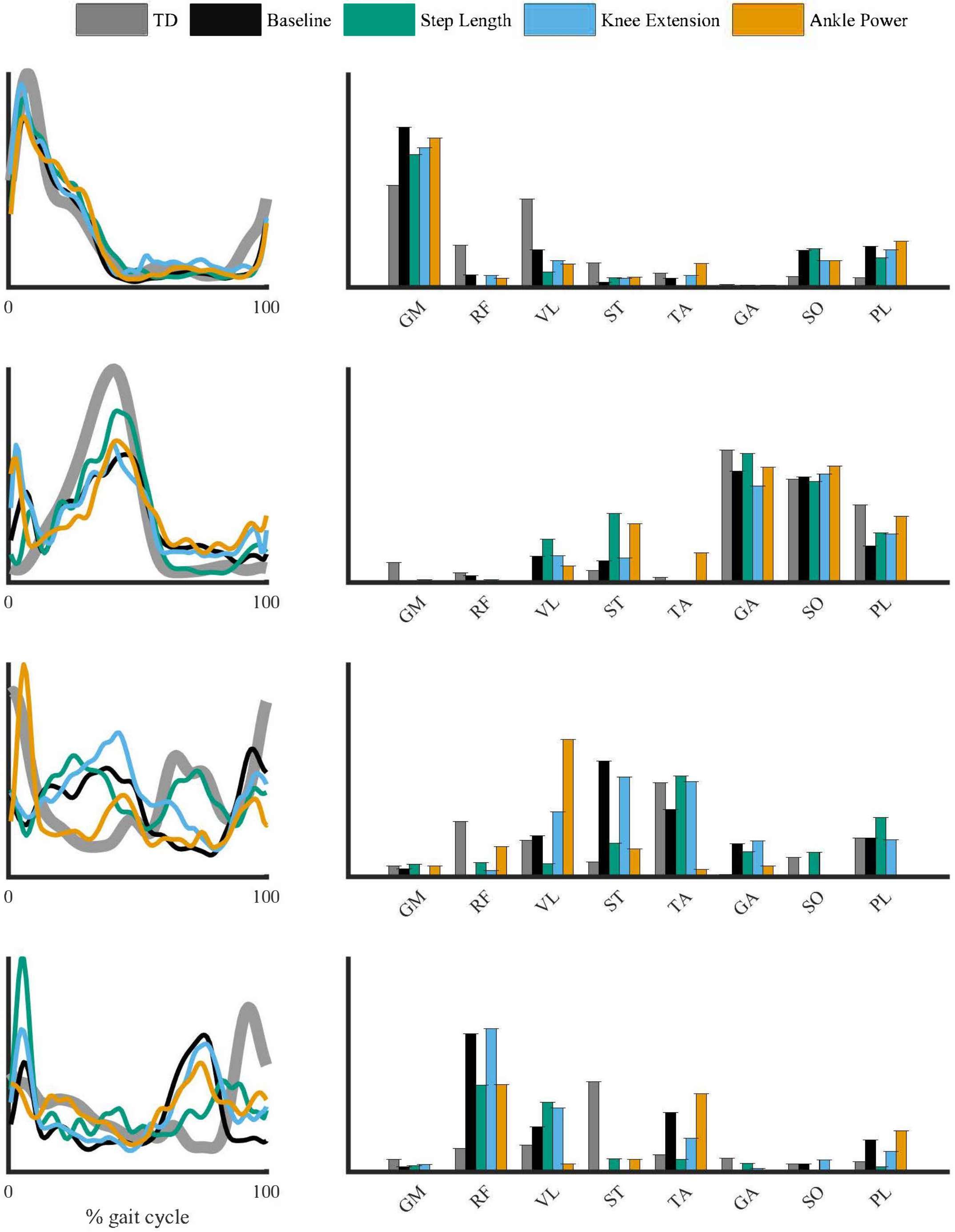

*Low-responder to biofeedback

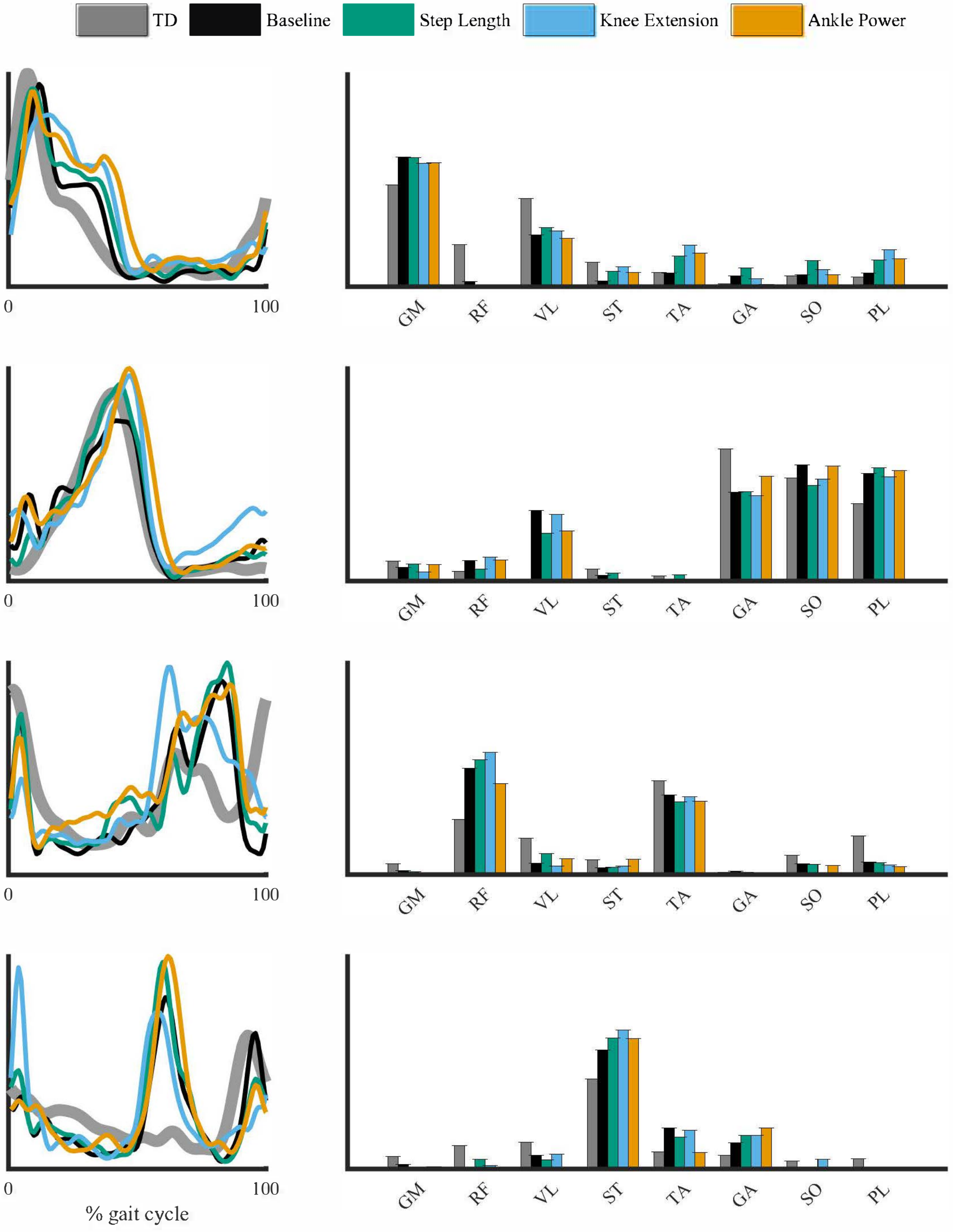

***High-responder to biofeedback**

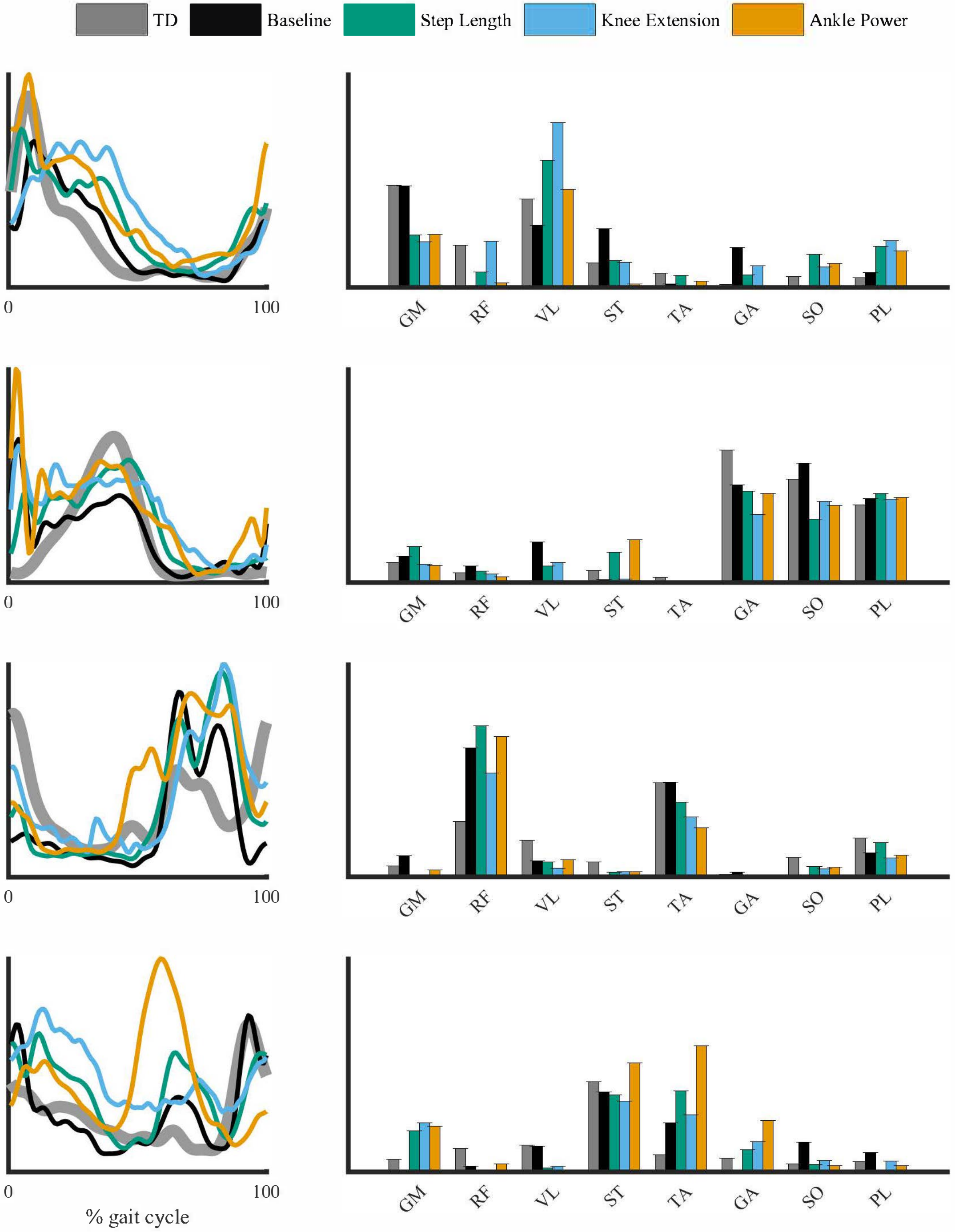

*Low-responder to biofeedback

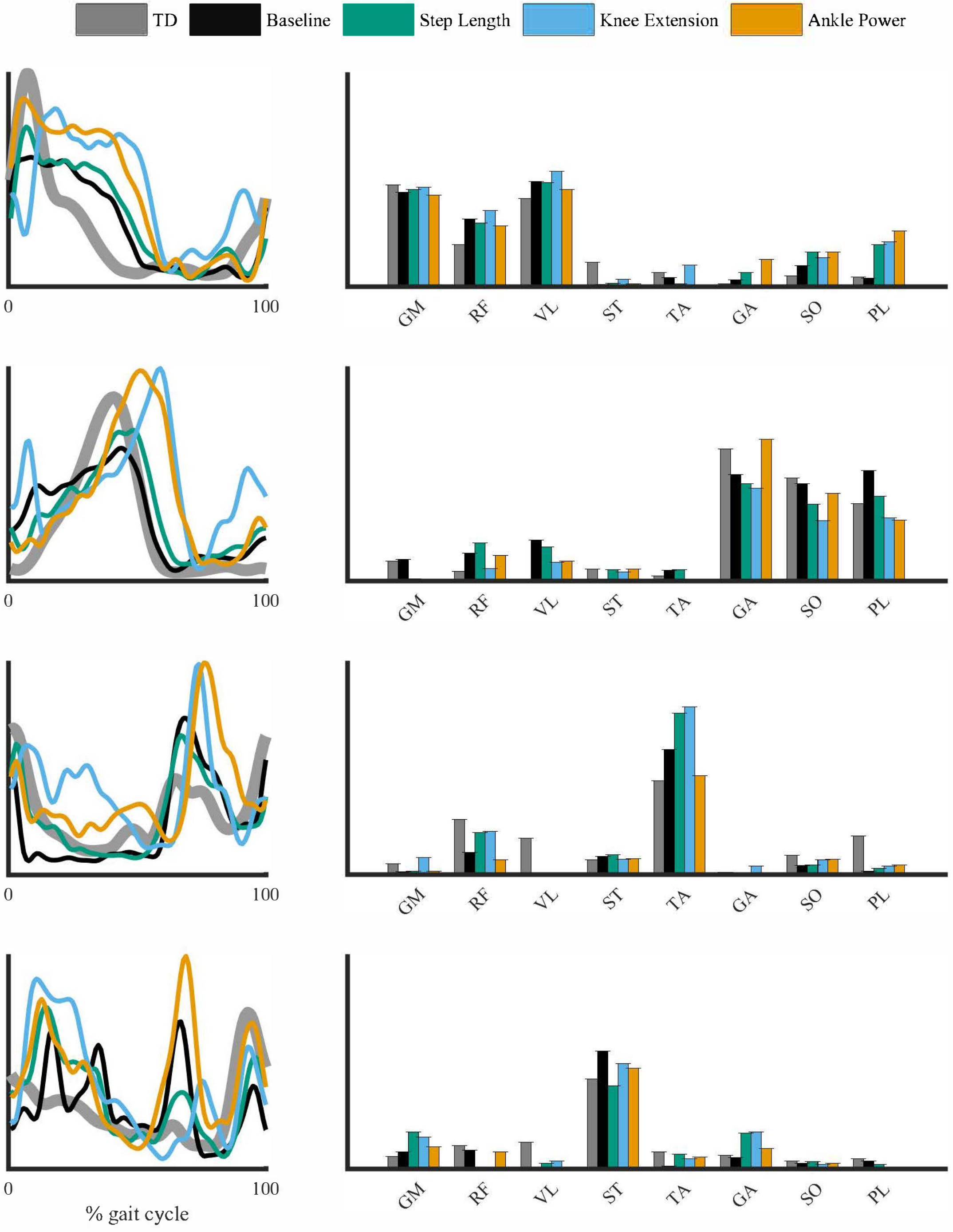

*High-responder to biofeedback

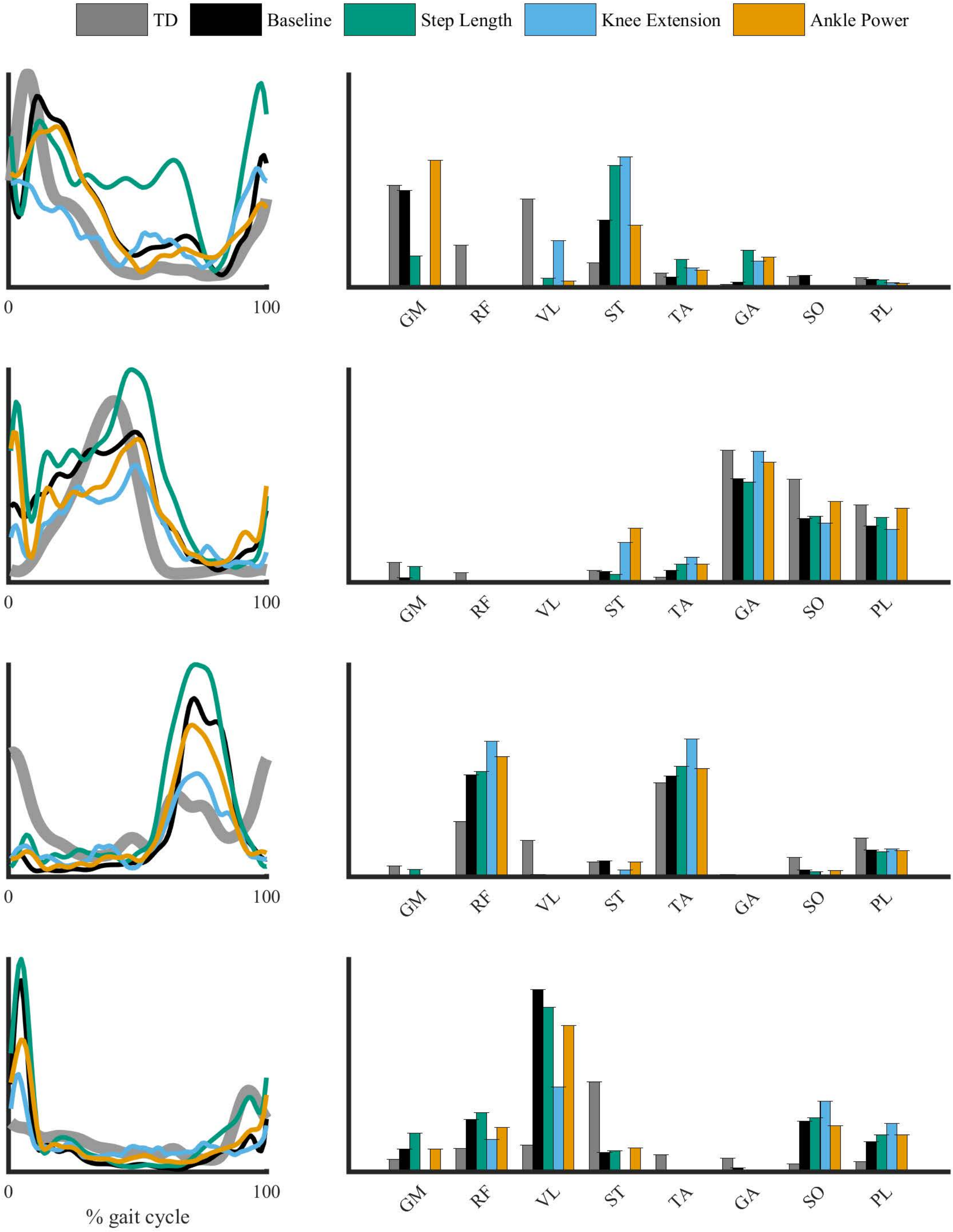
TD
Baseline
Step Length
Knee Extension
Ankle Power
0
100
GM
RF
VL
ST
TA
GA
SO
PL
% gait cycle

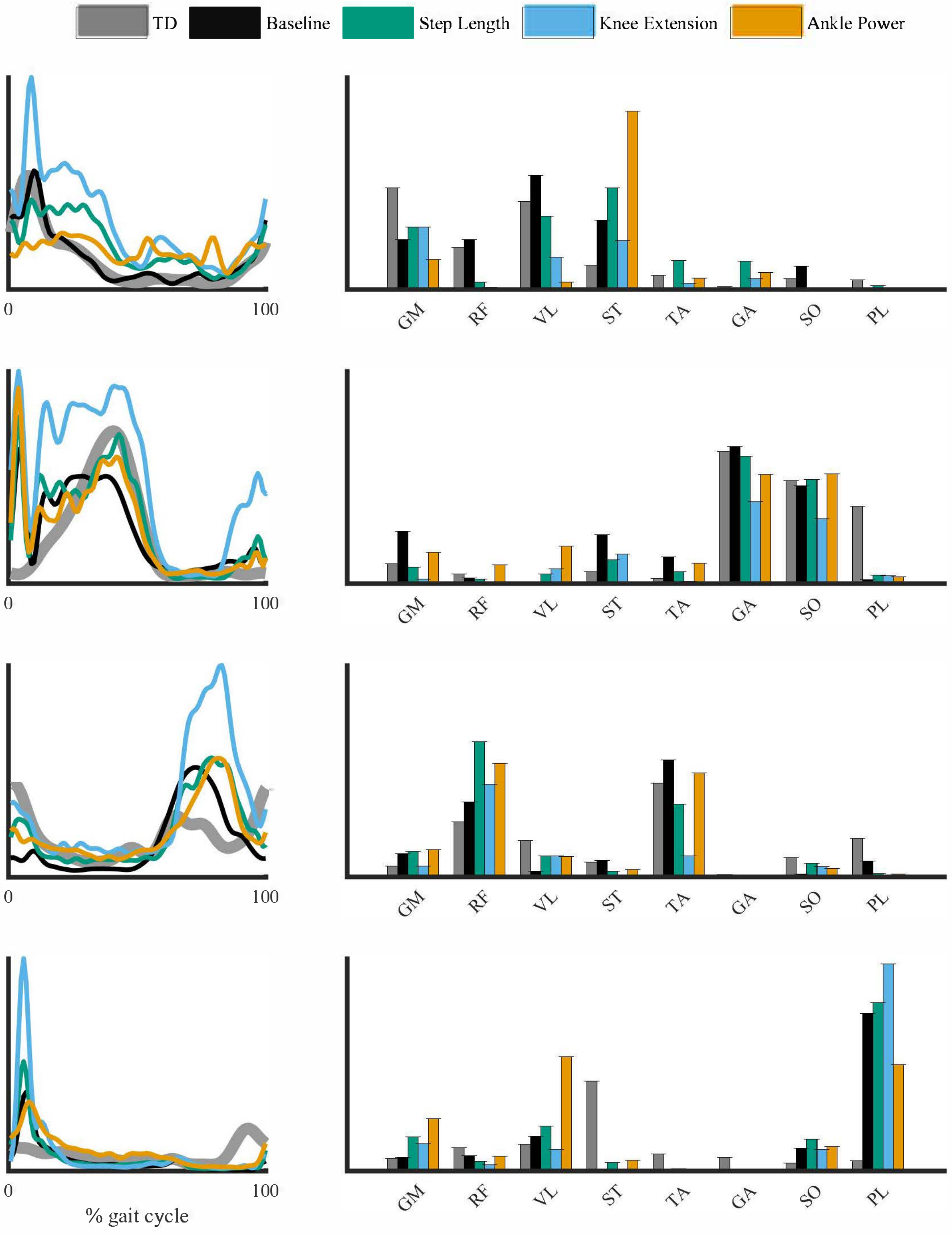

*High-responder to biofeedback

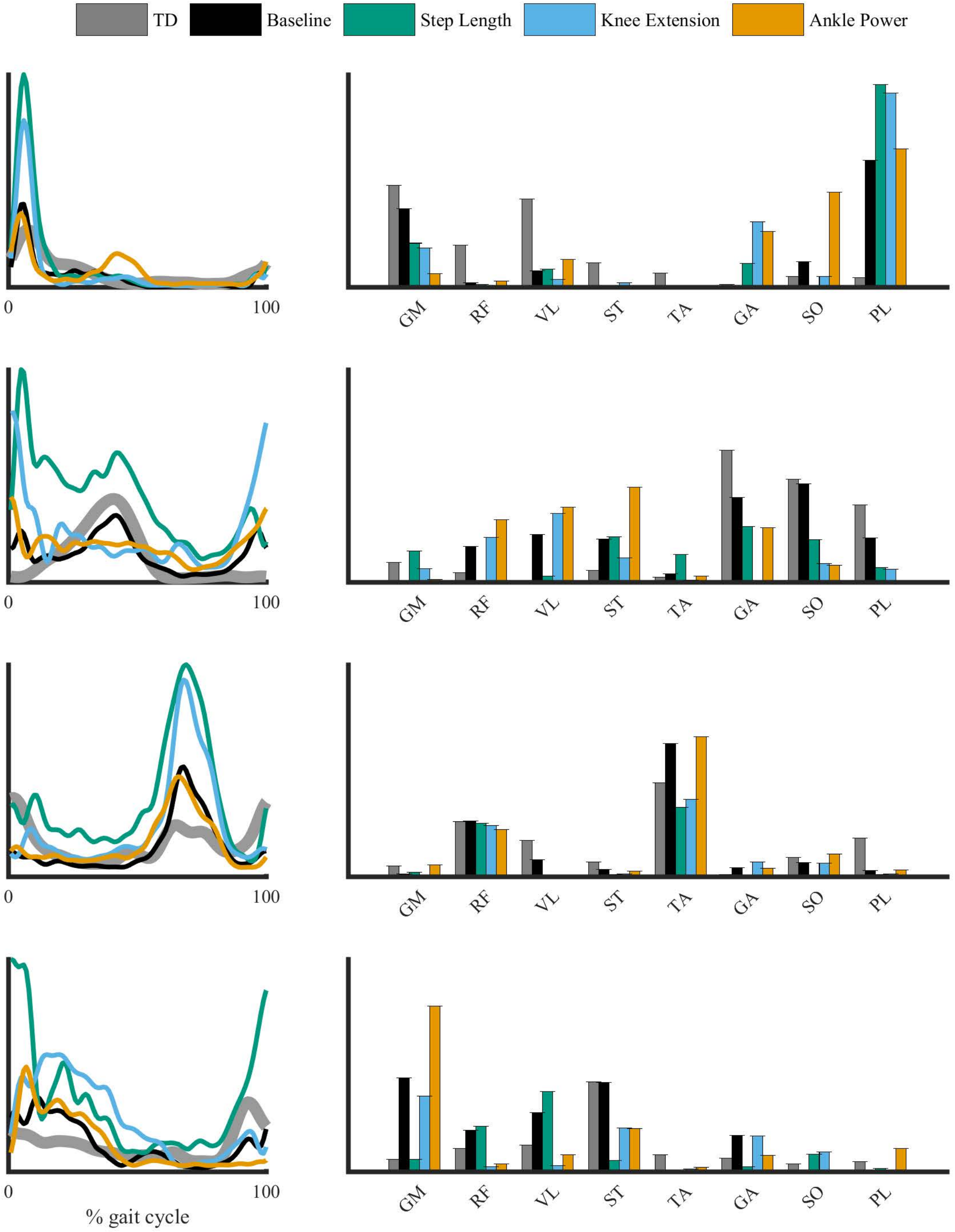
TD
Baseline
Step Length
Knee Extension
Ankle Power
0
100
GM
RF
VL
ST
TA
GA
SO
PL
% gait cycle

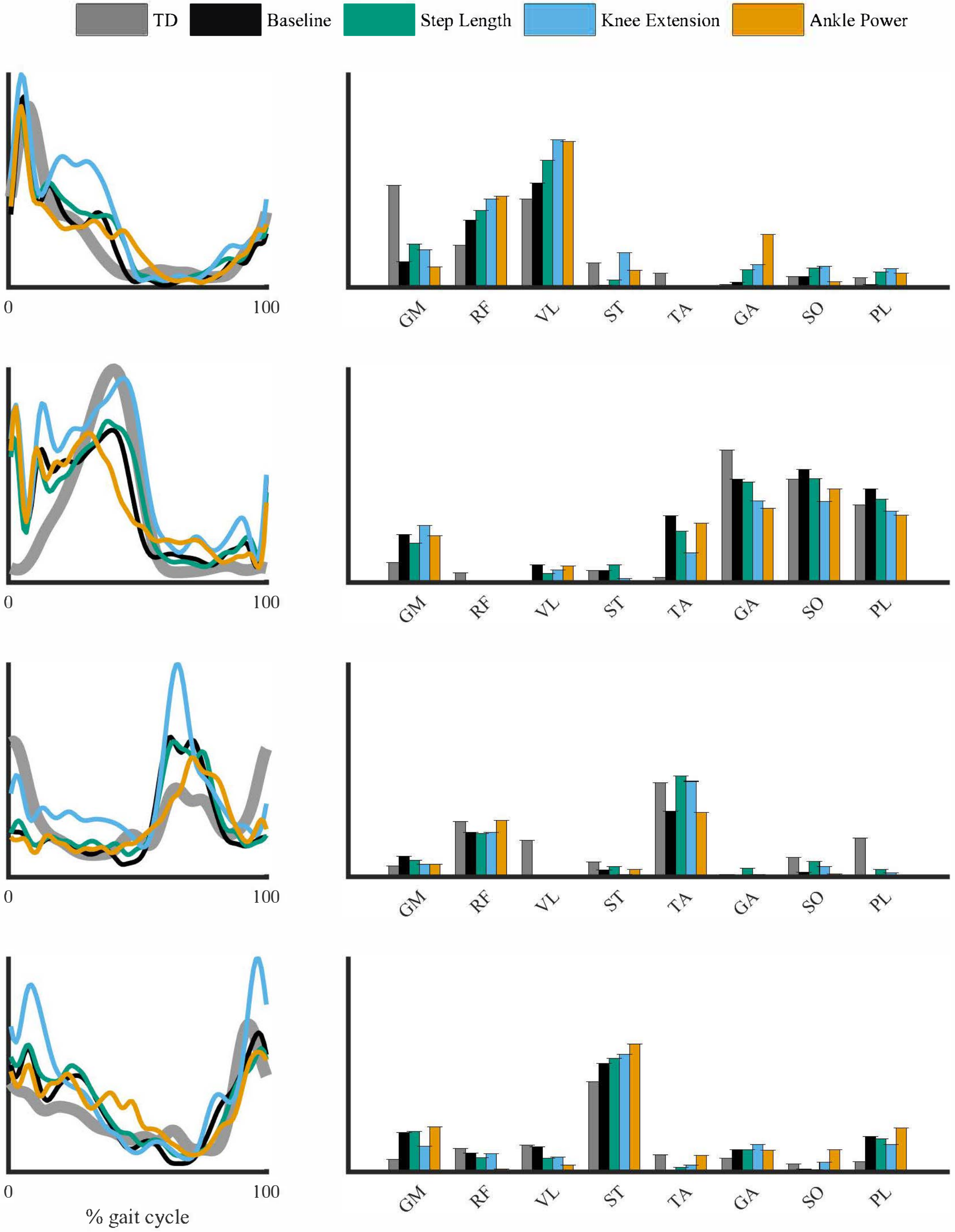

*Low-responder to biofeedback

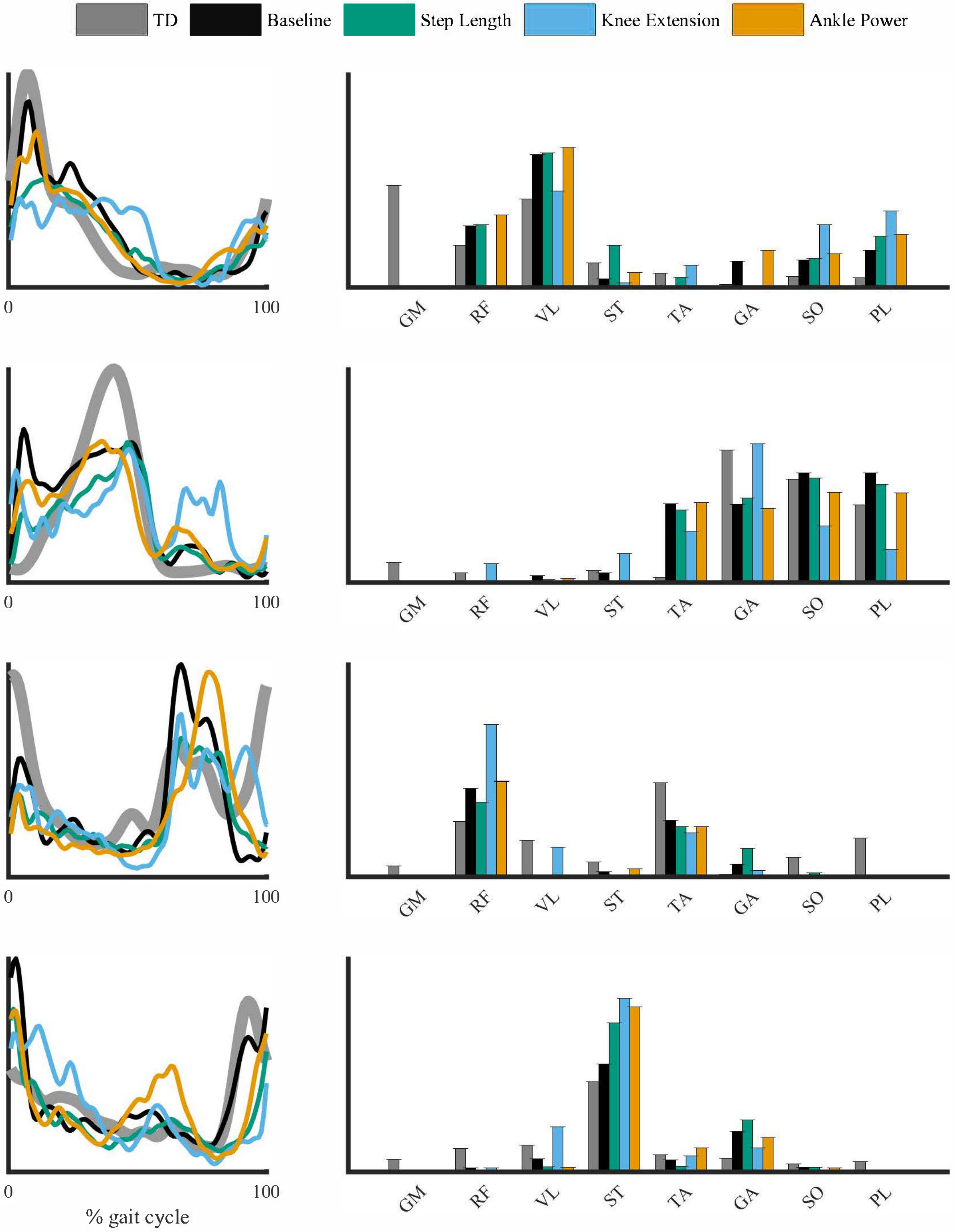

*Low-responder to biofeedback

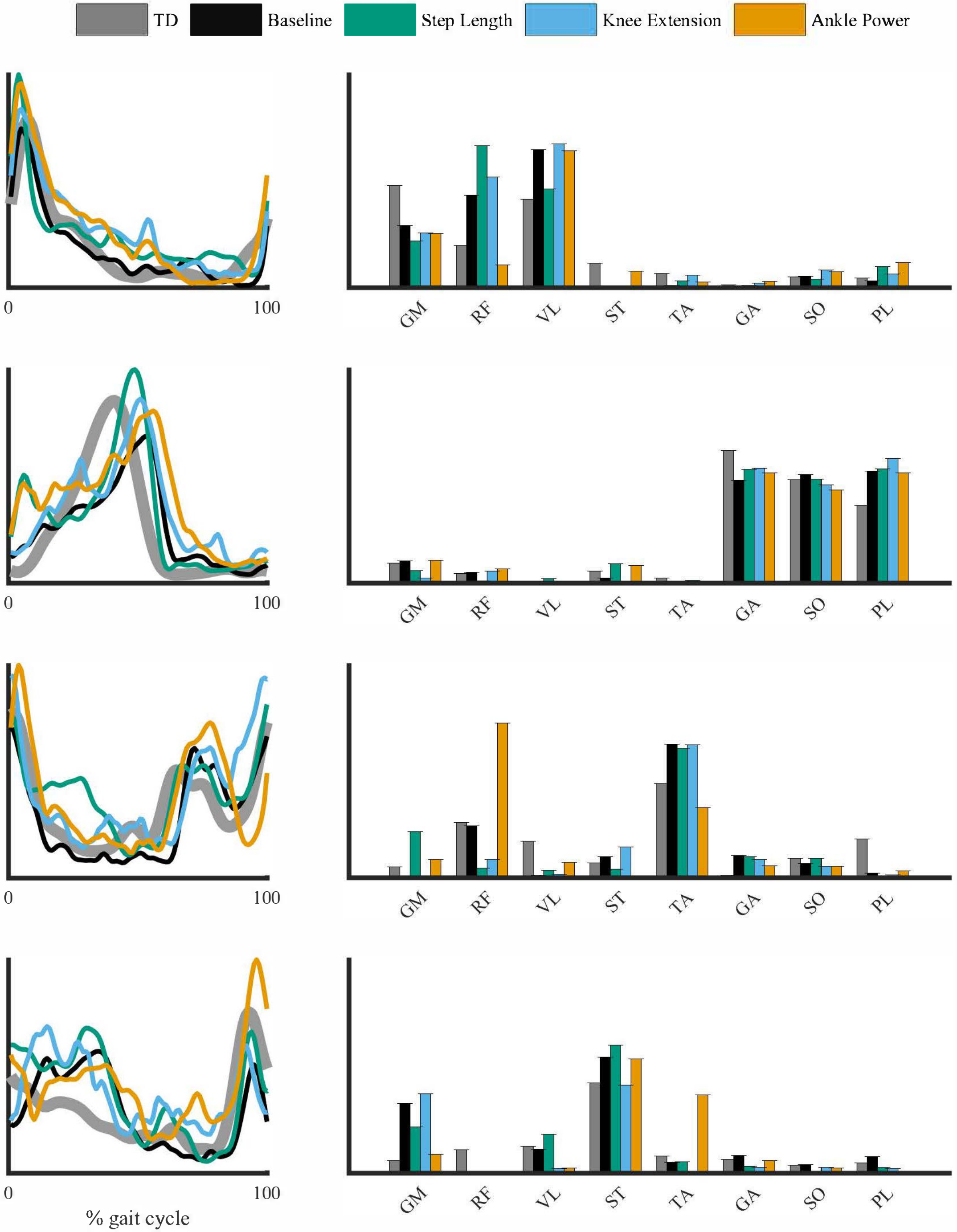

*Low-responder to biofeedback

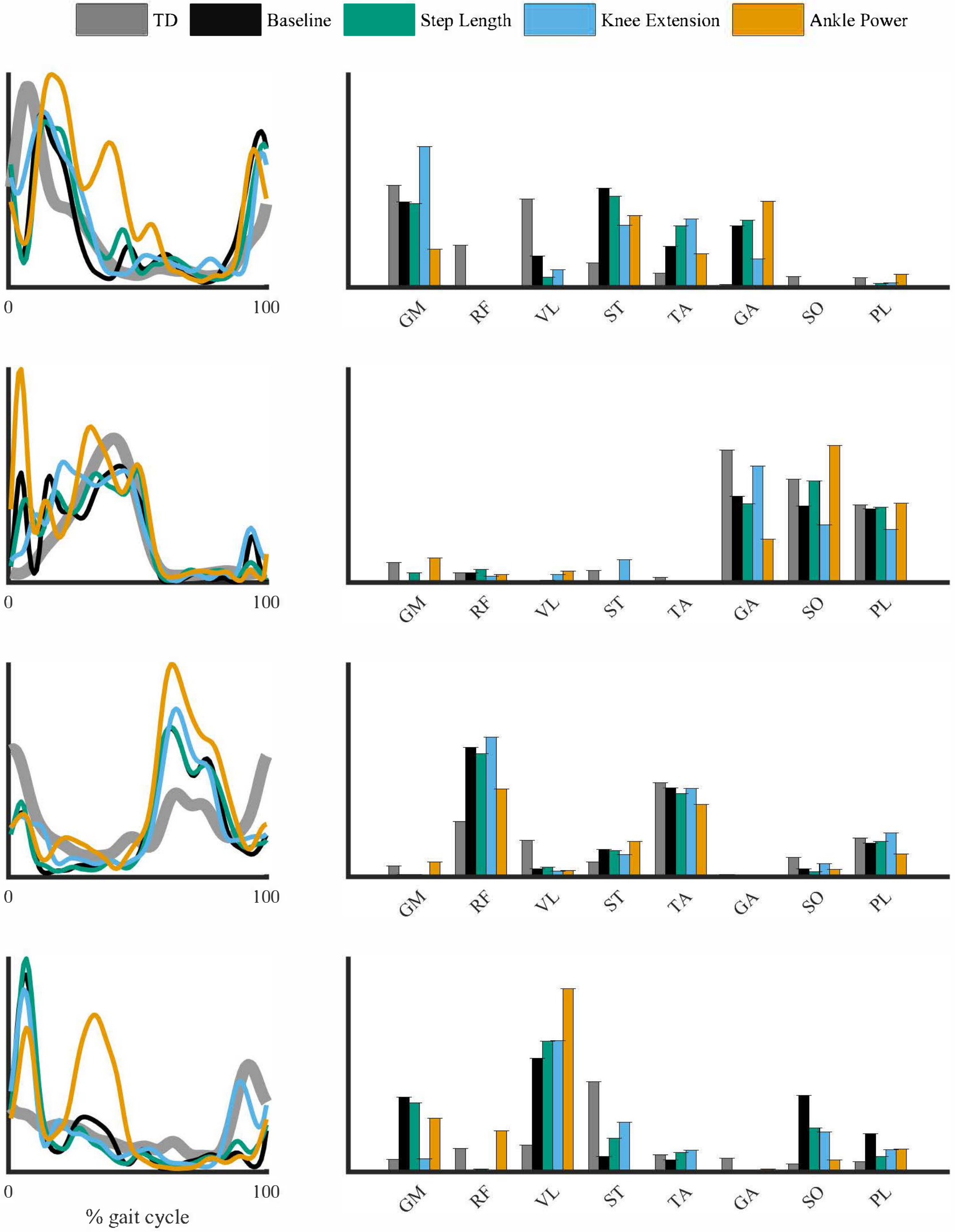

*High-responder to biofeedback

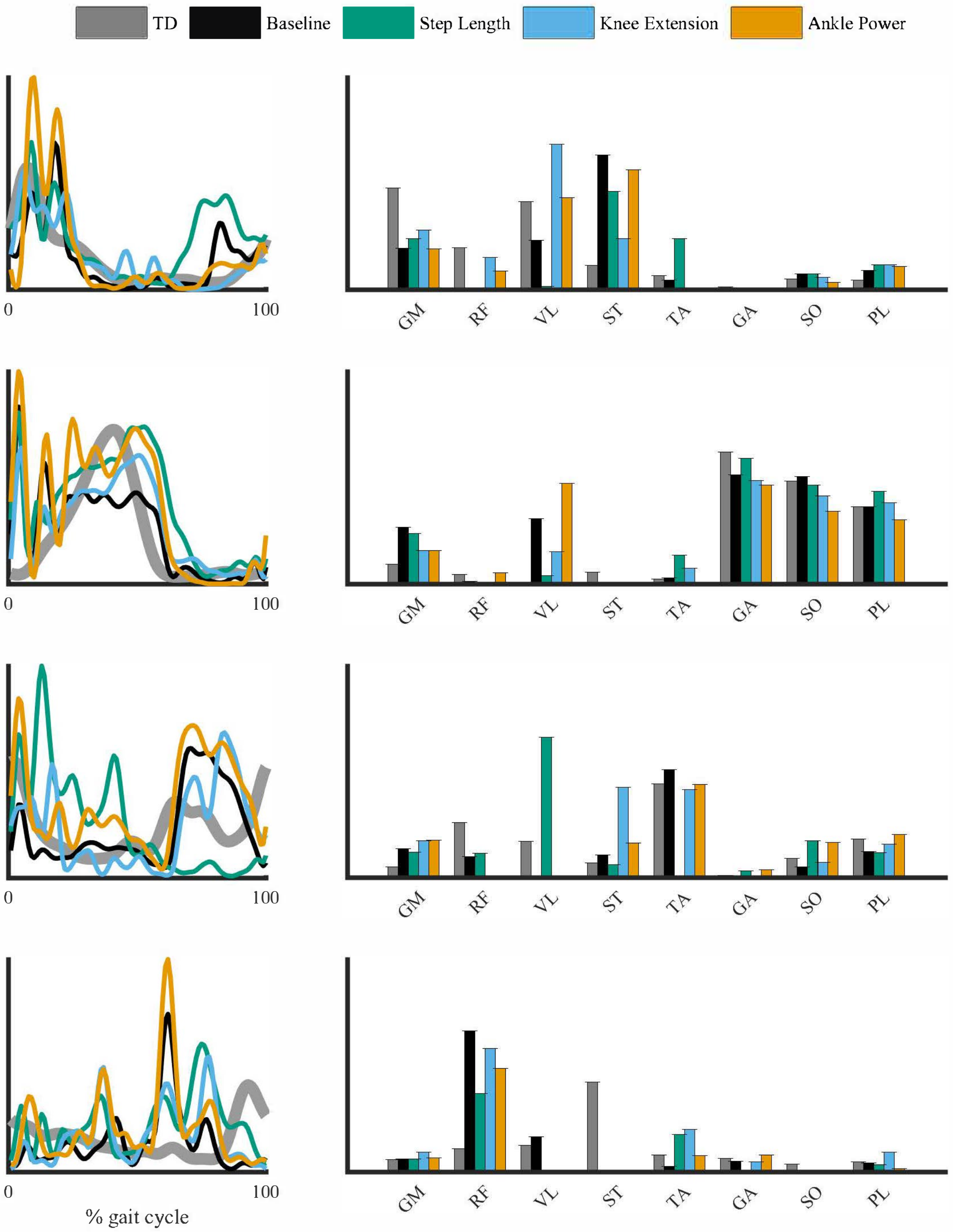

*Low-responder to biofeedback

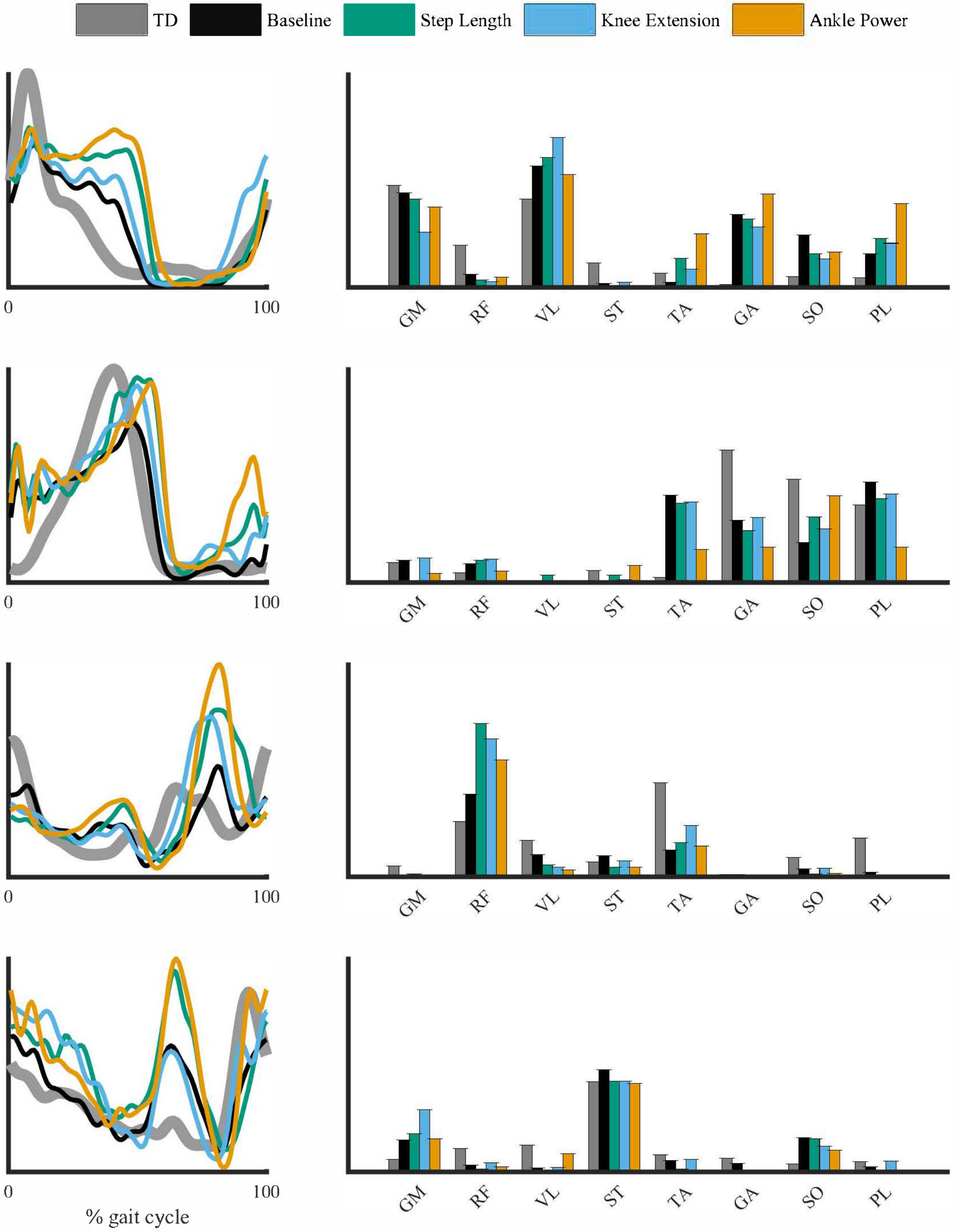

*High-responder to biofeedback

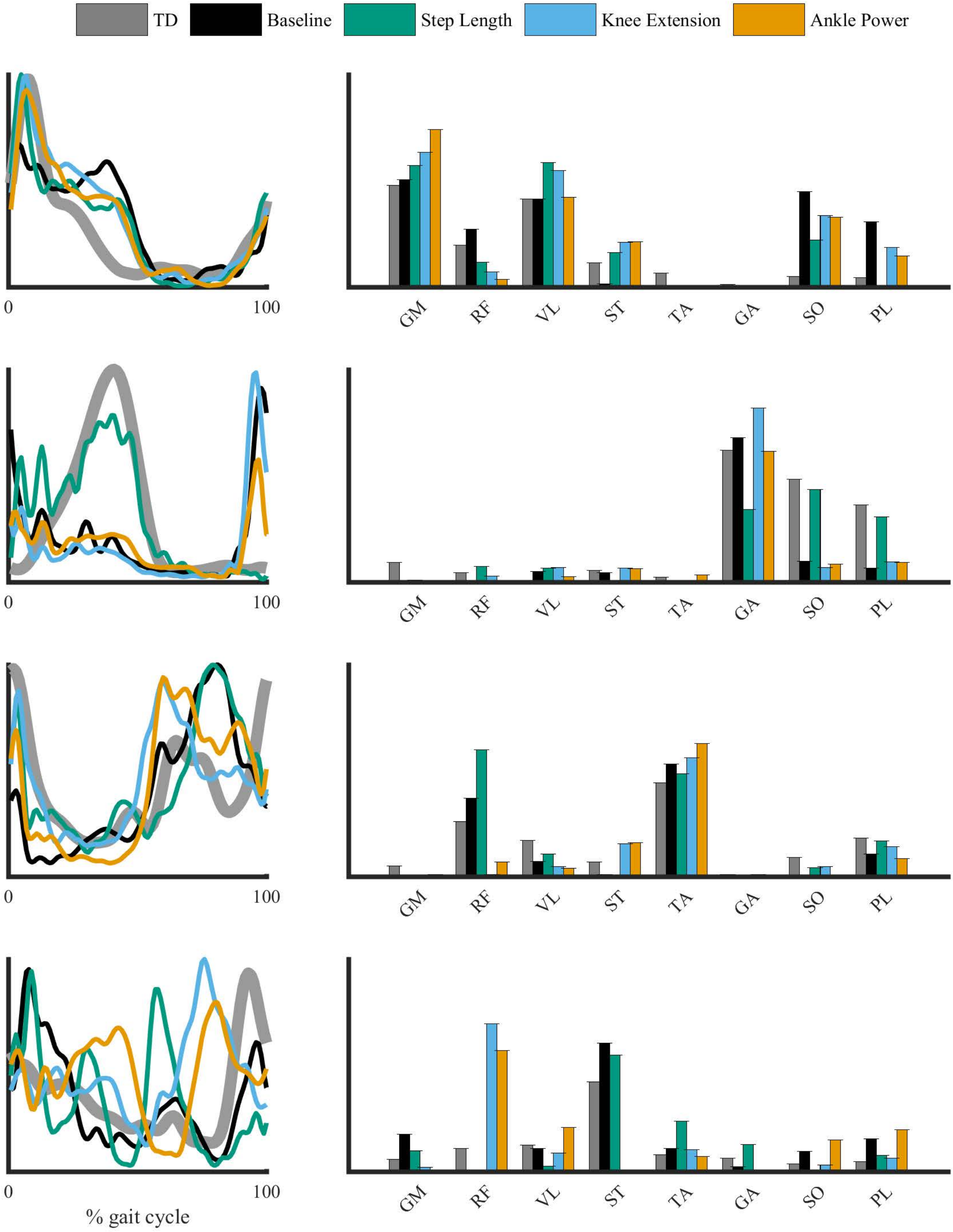
TD
Baseline
Step Length
Knee Extension
Ankle Power
0
100
GM
RF
VL
ST
TA
GA
SO
PL
% gait cycle

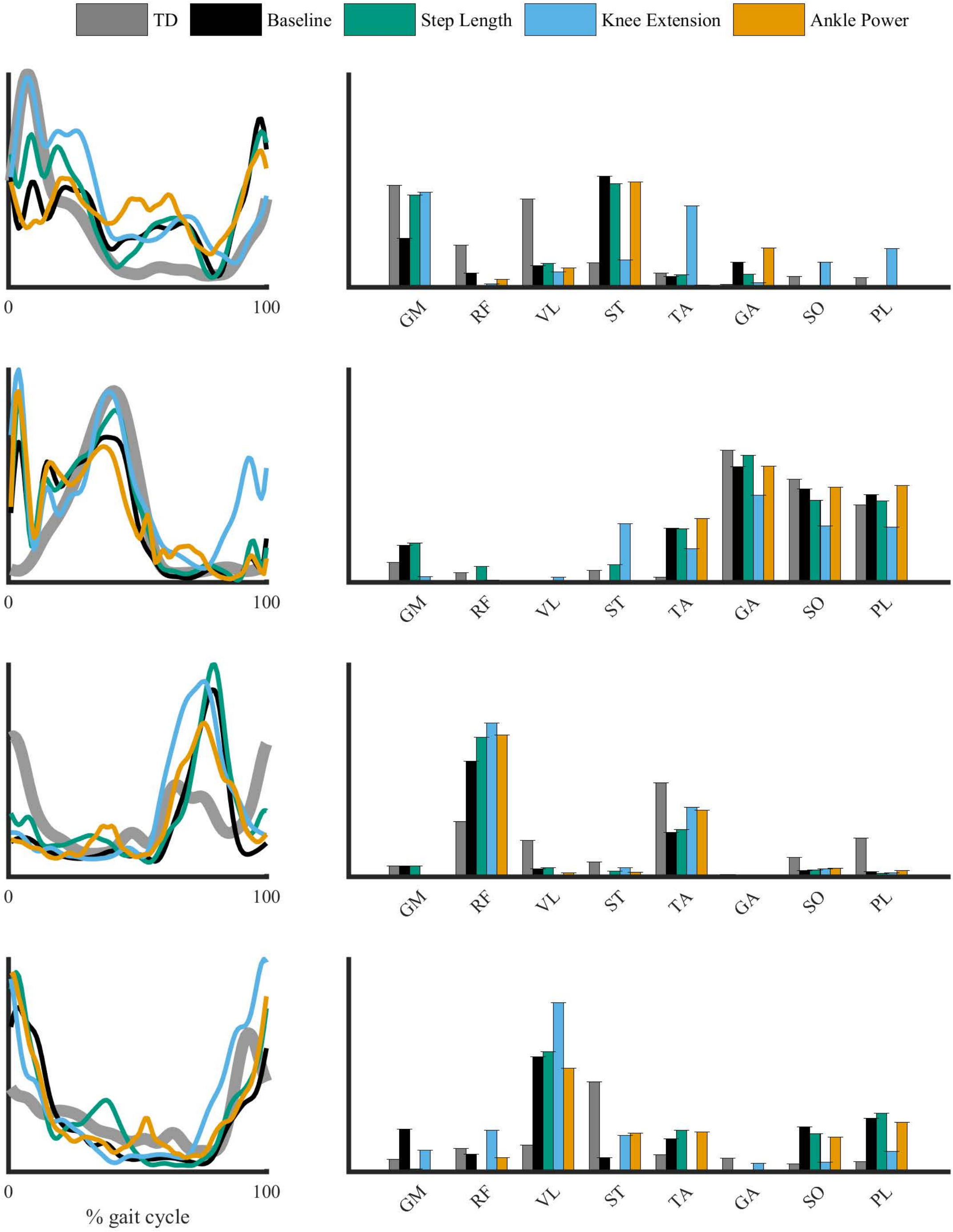

TD
Baseline
Step Length
Knee Extension
Ankle Power
0
100
GM
RF
VL
ST
TA
GA
SO
PL
% gait cycle

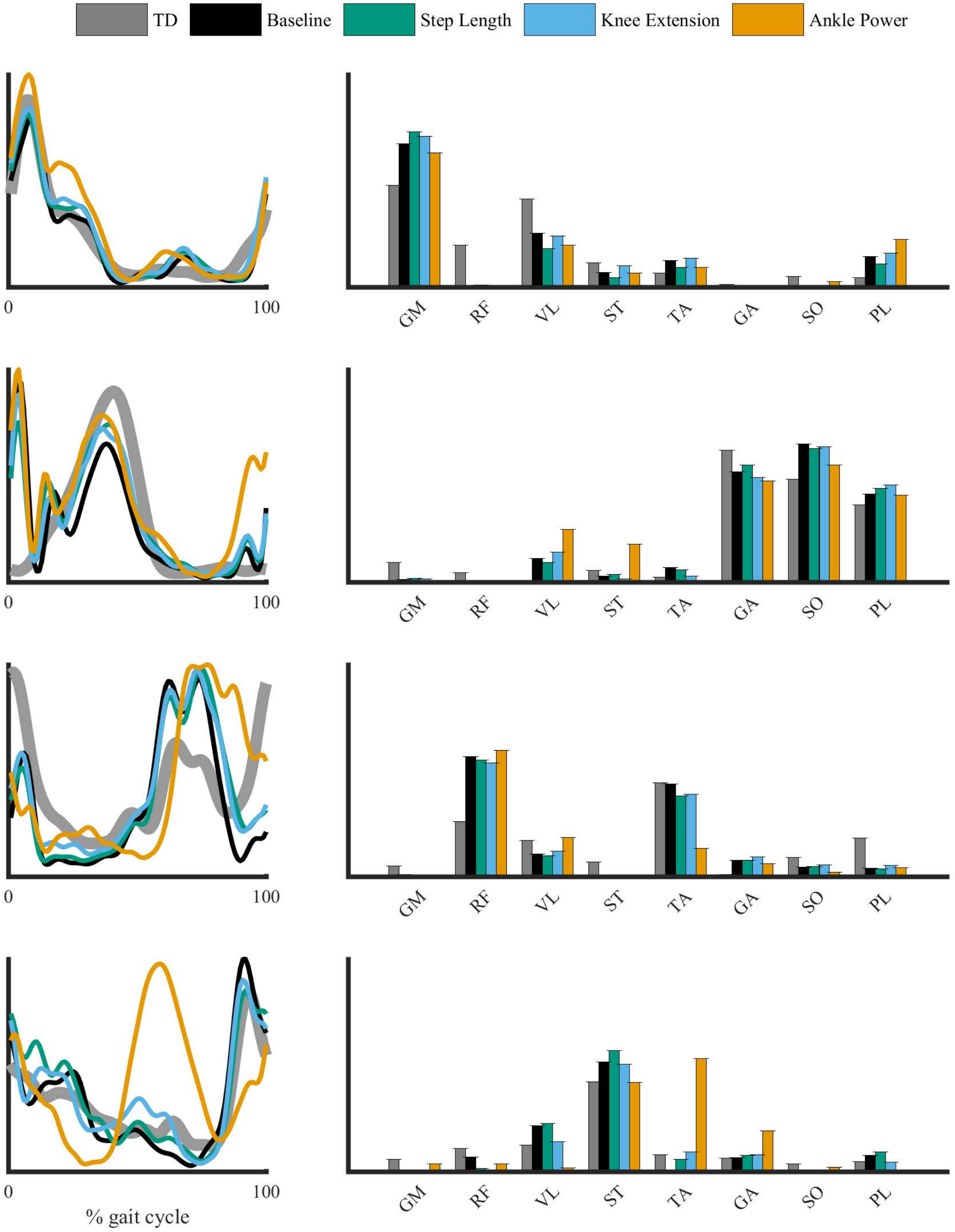
TD
Baseline
Step Length
Knee Extension
Ankle Power
0
100
GM
RF
VL
ST
TA
GA
SO
PL
% gait cycle
