# Supplementary material for: Muscle Synergies in Response to Biofeedback-Driven Gait Adaptations in Children With Cerebral Palsy
Source: Front Physiol. 2019 Sep 27;10:1208. doi: 10.3389/fphys.2019.01208 (PMC6776587; doi:10.3389/fphys.2019.01208)
Supplement: Supplementary file 2 [file Data_Sheet_2.pdf]

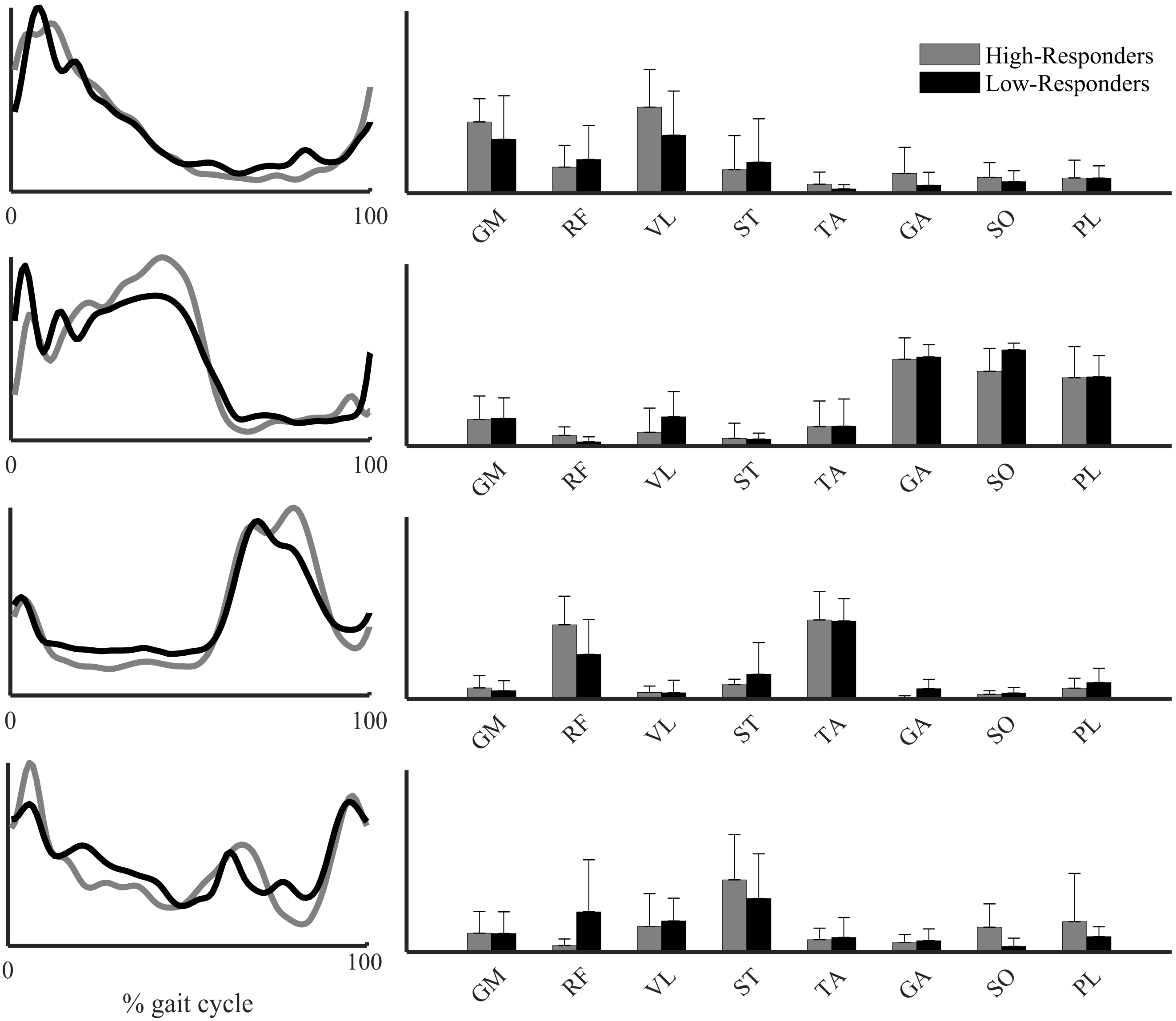


**Supplementary 2**. Comparison of high responders and low responders grouped response in synergy timing pattern (left) for the four-synergy solution. Corresponding muscle weightings (right) of each synergy pattern across feedback trials. Gluteus medius (GM), rectus femoris (RF), vastus lateralis (VL), semitendinosus (ST), tibialis anterior (TA), gastrocnemius medialis (GA), soleus (SO), and peroneus longus (PL).
